# TNF-mediated neuroinflammation is linked to neuronal necroptosis in Alzheimer's disease hippocampus

**DOI:** 10.1186/s40478-021-01264-w

**Published:** 2021-09-28

**Authors:** Anusha Jayaraman, Thein Than Htike, Rachel James, Carmen Picon, Richard Reynolds

**Affiliations:** 1 Centre for Molecular Neuropathology, Lee Kong Chian School of Medicine, Nanyang Technological University, Singapore, Singapore; 2 Division of Neuroscience, Department of Brain Sciences, Faculty of Medicine, Imperial College London, London, UK

**Keywords:** Necroptosis, Alzheimer’s disease, Post-mortem brain, iPSC, Tumor necrosis factor, ESCRT III

## Abstract

The pathogenetic mechanisms underlying neuronal death and dysfunction in Alzheimer’s disease (AD) remain unclear. However, chronic neuroinflammation has been implicated in stimulating or exacerbating neuronal damage. The tumor necrosis factor (TNF) superfamily of cytokines are involved in many systemic chronic inflammatory and degenerative conditions and are amongst the key mediators of neuroinflammation. TNF binds to the TNFR1 and TNFR2 receptors to activate diverse cellular responses that can be either neuroprotective or neurodegenerative. In particular, TNF can induce programmed necrosis or necroptosis in an inflammatory environment. Although activation of necroptosis has recently been demonstrated in the AD brain, its significance in AD neuron loss and the role of TNF signaling is unclear. We demonstrate an increase in expression of multiple proteins in the TNF/TNF receptor-1-mediated necroptosis pathway in the AD post-mortem brain, as indicated by the phosphorylation of RIPK3 and MLKL, predominantly observed in the CA1 pyramidal neurons. The density of phosphoRIPK3 + and phosphoMLKL + neurons correlated inversely with total neuron density and showed significant sexual dimorphism within the AD cohort. In addition, apoptotic signaling was not significantly activated in the AD brain compared to the control brain. Exposure of human iPSC-derived glutamatergic neurons to TNF increased necroptotic cell death when apoptosis was inhibited, which was significantly reversed by small molecule inhibitors of RIPK1, RIPK3, and MLKL. In the post-mortem AD brain and in human iPSC neurons, in response to TNF, we show evidence of altered expression of proteins of the ESCRT III complex, which has been recently suggested as an antagonist of necroptosis and a possible mechanism by which cells can survive after necroptosis has been triggered. Taken together, our results suggest that neuronal loss in AD is due to TNF-mediated necroptosis rather than apoptosis, which is amenable to therapeutic intervention at several points in the signaling pathway.

**Supplementary Information:**

The online version contains supplementary material available at 10.1186/s40478-021-01264-w.

## Introduction

In recent years, accumulating evidence suggests that the longstanding amyloid cascade hypothesis cannot sufficiently explain many aspects of Alzheimer’s disease (AD) pathogenesis, bringing the exploration of other possible underlying mechanisms to the forefront [32]. Neuroinflammation is suggested to play a major role in ongoing neurodegeneration in AD, as demonstrated by increased inflammatory markers in patients with AD, the discovery of AD risk genes associated with innate immunity [21], and the prominent presence of dysregulated immune pathways in the genomic and transcriptomics analyses of AD tissues [72]. One of the key mediators of inflammation in many systemic chronic inflammatory and degenerative conditions is the soluble form of tumor necrosis factor (TNF), acting primarily through its binding to TNF receptor 1 (TNFR1) [12], and there is increasing evidence that TNF signaling is also implicated in multiple neurodegenerative conditions [71]. TNF binding to the death domain containing TNFR1, and subsequent formation of complex 1, can lead to either induction of NFκB signaling or cytotoxicity via apoptosis or necroptosis following formation of complex 2. Apoptosis is triggered when the receptor interacting kinase 1 (RIPK1) is deubiquitinated by CYLD protein and then cleaved by activated caspase 8 [40, 42]. However, under chronic inflammatory conditions characterized by down-regulated caspase 8 activation, RIPK1 interacts with RIPK3 to induce autophosphorylation, leading to the recruitment and phosphorylation of pseudokinase mixed lineage kinase domain-like (MLKL), which together form the necrosome. pMLKL oligomers then translocate to the plasma membrane and disrupt it, leading to necroptotic cell death [60, 65].

Apoptotic neurons are only very rarely detected in the AD brain [26], whereas necroptosis has been recently implicated in several neurodegenerative and neuroinflammatory disorders, including multiple sclerosis (MS) [43, 46], AD [5, 30], and amyotrophic lateral sclerosis [48], but the triggering and signaling mechanisms underlying necroptotic cell death in these conditions are still unclear. There are a number of known triggers of necroptotic signaling in non-neuronal cells, of which TNF is the most studied [76]. Although TNF is suggested to be a key mediator of chronic inflammation and cytotoxicity in many neurodegenerative and neuroinflammatory disorders [71], few comprehensive human tissue studies have been undertaken to investigate the signaling pathways concerned and the cellular specificity. Levels of TNF in the cerebrospinal fluid (CSF) have been shown to be increased in AD at an early stage [23, 61, 62] and TNF and TNFR1 protein levels have been reported to be increased in early-stage AD post-mortem brains [75]. Treatment of primary microglial cultures with Aβ has been shown to result in high levels of TNF release from these cells [8]. Chronic neuron-specific expression of TNF in 3xTg-AD mice has been shown to result in inflammation-driven neuronal death in this AD mouse model [25]. In addition, a single nucleotide polymorphism in the TNF gene, G308A, has been reported to have a significant association with susceptibility to AD in certain populations, while being protective in others [67]. Our previous studies on neurodegeneration in the MS brain indicate that, in this prototypical neuroinflammatory condition, TNF/TNFR1 interaction and downstream activation of the RIPK1/RIPK3/MLKL kinase cascade is the most likely cause of neuronal necroptosis [46]. Here we show the activation of the TNFR1-mediated necroptosis pathway in hippocampal CA1-2 neurons and a concomitant downregulation of apoptotic signaling in a cohort of post-mortem AD brains. In addition, we report a dysregulation in the ESCRTIII pathway, which has been suggested to be involved in the rescue of cells from necroptosis in non-CNS systems [16]. Treatment of human iPSC-derived glutamatergic neurons with TNF led to necroptotic cell death when apoptosis was inhibited. Small molecule inhibitors of RIPK1, RIPK3 and MLKL protected these neurons against TNF-mediated cell death, suggesting new potential therapeutic avenues against neurodegeneration in the AD brain.

## Materials and methods

### Tissue samples

The AD post-mortem tissues for this study were obtained from the Multiple Sclerosis and Parkinson’s Tissue Bank at Imperial College London and the South West Dementia Brain Bank, University of Bristol. Snap frozen tissue blocks of anterior hippocampus and entorhinal cortex were obtained from 30 AD cases (12 males, 18 females; median age of death = 85.5 years, range = 57‒99 years; Braak stages III‒VI) and 11 control cases (6 males, 5 females; median age of death = 83 years, range 63‒95 years). Formalin fixed paraffin embedded (FFPE) sections were obtained for a subset of cases and controls (n = 5 per group) from the Multiple Sclerosis and Parkinson’s Tissue Bank at Imperial College London. Fully informed consent was obtained for the post-mortem donation under ethical approval by the National Research Ethics Committee (08/MRE09/31 and NHS REC No 18/SW/0029). The project was approved by the Nanyang Technological University Institutional Review Board (IRB-2018-09-052). The demographic data and neuropathological features of the AD cases provided by both brain banks were determined in accordance with the standardized criteria of the UK MRC Brain Bank Network and are shown in Additional file 7: Table 1.

### Immunohistochemistry and immunofluorescence

For immunofluorescence analysis, 10 µm cryosections containing the CA1 region were cut from the snap frozen hippocampal blocks. For immunohistochemistry, snap frozen tissue sections were fixed with formalin for 30 min followed by ice cold methanol for 10 min. Slides were blocked with 10% normal goat or horse serum followed by overnight incubation with primary antibody at 4 °C and 30 min incubation with ImmPRESS HRP-conjugated secondary antibodies (Vector Laboratories) at room temperature. Slides were visualized with ImmPACT-DAB (Vector Laboratories) as the chromogen. Sections were counterstained with hematoxylin and DPX (Sigma-Aldrich) mounted. Dual color IHC was performed sequentially. Detection of the primary antibody with ImmPACT-DAB was followed by incubation with the second primary antibody, which was detected using the ABC-alkaline phosphatase detection system (Vector Laboratories), using Vector blue as the substrate. When using human paraffin embedded sections for IHC, the sections were deparaffinized and subjected to heat-induced epitope retrieval using citrate buffer before following the same steps as for snap-frozen tissue. For immunofluorescence, 10 µm human tissue cryosections were fixed with ice cold methanol, blocked with 10% normal goat or horse serum, and incubated overnight with primary antibodies. Sections were then incubated with the appropriate secondary antibody conjugated to a fluorochrome and mounted with Vectashield® Vibrance™ Antifade Mounting medium with DAPI (Vector Laboratories) for nuclei counterstaining. Individual antibody details are listed in Additional file 8: Table 2.

### Protein extraction

Hippocampal grey matter from snap-frozen human AD and control samples was dissected by carefully scoring the tissue block with a fine scalpel and subsequent isolation of the tissue by cryosectioning in a Leica cryostat. The resultant tissue samples were homogenized in RIPA buffer (ab156034, Abcam) containing protease and phosphatase inhibitors (ab201119, Abcam) and incubated on ice for 20 min at 4 °C. The protein extract was centrifuged at 14,800 g for 15 min at 4 °C. The resulting supernatant was taken as the RIPA soluble fraction. Pellets were washed in TBS and homogenized in 6 M urea/5% SDS for 30 min at room temperature (RT) and centrifuged at 14,800 g for 10 min at 4 °C. The resultant supernatant was the urea fraction. The samples were stored at − 80 °C until further use. To extract proteins in native condition, tissue was homogenized in TBS containing 0.1% of Triton X-100, incubated for 10 min at RT, followed by centrifugation at 16,000 g for 10 min.

### Western blotting

Briefly, protein concentration was quantified using a Sigma-Aldrich BCA protein assay kit (Merck). Subsequently, 10–50 μg of protein was loaded onto 4–15% Mini-PROTEAN® TGX Stain-Free™ Protein Gels (Bio-Rad) and transferred to polyvinylidene difluoride (PVDF) membranes for 40–60 min. The membranes were incubated for 1 h in 5% BSA at room temperature and incubated overnight at 4 °C with the appropriate primary antibodies. The blots were then washed 3 × with TBS-0.1% Tween 20 (TBS-T) for 10 min each and incubated with the horseradish peroxidase (HRP) conjugated specific secondary antibodies (1:20,000, Bio-Rad) for 1 h at RT. The blots were then washed with TBS-T, and imaged/quantified using a Bio-Rad ChemiDoc™ MP Imaging System and normalized with GAPDH expression. For the detection of MLKL oligomers, proteins were run on 7.5% Mini-PROTEAN® TGX Stain-Free™ Protein Gel (Bio-Rad) in non-reducing conditions. Individual antibodies are listed in Additional file 8: Table 2.

### RNA extraction and RT-PCR

For RNA extraction, grey matter regions in each brain tissue block were demarcated with a scalpel and then cryosectioned as above. The grey matter tissue sections were then homogenized and processed for total RNA extraction using PureLink™ RNA Mini kit (Life Technologies Corporation) as per the manufacturer’s protocol. Purified total RNA (100 ng) was used from each sample for One-step reverse transcriptase quantitative polymerase chain reaction (RT-qPCR) using the iTaq™ Universal SYBR® Green One-Step kit (Bio-Rad) in the StepOnePlus™ Real Time PCR system (Applied Biosystems). All primers in the study were commercially purchased PrimePCR™ SYBR® Green assay primers (Bio-Rad). For each sample, reactions were set up in triplicate with the following cycling protocol: 50 °C for 10 min, 95 °C for 1 min, 40 cycles with a 3-step protocol (95 °C for 15 s, 60 °C for 1 min), and a final melting curve analysis with a ramp from 65 to 95 °C. Relative quantification of mRNA levels from various treated samples was determined by the comparative Ct method [54] after normalizing with the corresponding *xpnpep1* levels [9] from the samples.

### Immunoprecipitation

Samples from AD and control brain grey matter were homogenized in RIPA buffer containing protease and phosphatase inhibitors. Immunoprecipitation was carried out using the Dynabeads™ Protein G Immunoprecipitation Kit (ThermoFisher Scientific) according to the manufacturer’s protocol. Briefly, 10 μg of anti-MLKL antibody was incubated for 10 min with 50 µl of Dynabeads Protein G while rotating. The beads were then washed and incubated with control and AD lysate samples overnight at 4 °C. The beads were then washed three times with washing buffer and resuspended in the elution buffer mixed with 2X sample buffer (Bio-Rad). The beads were boiled at 95 °C for 5 min and placed on ice. Bead-free supernatant samples were loaded onto a 4–15% Mini-PROTEAN® TGX Stain-Free™ Protein Gels. Gels were transferred to a PVDF membrane and incubated overnight at 4 °C with anti-pRIPK3 followed by 1 h incubation with anti-rabbit IgG-HRP secondary antibody at RT. Blots were then washed and developed with Amersham™ ECL™ Select WB Detection Reagent. Individual antibodies are listed in Additional file 8: Table 2.

### IPSC glutamatergic neuron culture

Human ioNEURONS/glut glutamatergic neurons (ab259259, Abcam) were seeded at 2 × 10^5^cell/well on 96 well plates (Biolab) coated with Geltrex in Neurobasal media supplemented with B27 Plus, GlutaMax™, penicillin/streptomycin, NT3 and BDNF (Life Technologies) as per the manufacturer’s protocol. The cultures were maintained in a humidified atmosphere of 5% CO_2_ in air at 37 °C. All experiments were performed at 9–11 days post-plating***.*** Treatment with TNF (ThermoFisher), SMAC mimetics (Tocris) and caspase inhibitor Z-VAD-FMK (Abcam) (TSZ treatment) was performed at the concentrations indicated in the text. Necroptosis inhibitors GSK-547 (SelleckChem), GSK-872 (Abcam) and necrosulfonamide (Abcam) were added to the cultures 1 h before the treatment with TSZ at concentrations indicated in the text.

### LDH assay

Cell cytotoxicity was determined by measuring the lactate dehydrogenase (LDH) release (ab65393, LDH-Cytotoxicity Assay kit, Abcam) from iPSC ioNEURONS/glut under different treatment conditions. Ten μl supernatant samples from the neuronal cultures under different conditions were collected and processed following the manufacturer’s protocol. The LDH released was determined by measuring the absorbance of the samples with a Synergy H1 microplate reader (BioTek) at 450 nm.

### Reactive oxygen species assay

A reactive oxygen species (ROS) assay was carried out using the Cellular ROS assay kit (ab133854, Abcam) according to the manufacturer’s protocol for adherent cells. Cells under different treatment conditions were incubated with the diluted 2′,7′-dichlorodihydrofluorescein diacetate (DCFDA) solution for 45 min at 37 °C in the dark. The solution was removed, and the cells were washed once with PBS. The culture plate was immediately measured on a fluorescence plate reader (Synergy H1, BioTek) at Ex/Em = 485/535 nm in endpoint mode. DCFDA is taken up by the cells, where in the presence of ROS, it is oxidized and converted to 2′,7′-dichlorofluorescein (DCF), which is highly fluorescent. ROS was quantified by measuring the fluorescence of DCF.

### Image acquisition and analysis

Immunohistochemistry slides were digitized by whole slide scanning using a Zeiss Axio Scan Z1 scanner. Image files were handled using QuPath v0.2.0. and ImageJ 1.53c open-source software for analyses. The entire CA1-2 hippocampus region was traced and measured in each slide/case before measuring HuC/D, pRIPK3 or pMLKL positive cell densities with the “Positive Cell Detection of a Region of Interest” tool in QuPath. For CD8 + T-cells, the entire CA1-2 region was demarcated for counting tissue-resident T-cells. For perivascular and meningeal T-cells, 5 regions of Interest (ROIs) around the hippocampus were selected in each slide and cells were counted manually. The total number of cells for all the experiments is reported as total cell density/mm^2^. The relative mean intensity of HLA-DR staining was measured with ImageJ software from 3 randomly chosen ROIs within the hippocampus for each slide. Immunofluorescence images from post-mortem tissues and iPSC cultures were captured using an Axio Observer 7 Inverted fluorescence microscope (Zeiss) at 40X magnification and processed with Zen 2 (Blue edition) software for analyses. For all immunofluorescence analyses, the settings for digital imaging were fixed during acquisition for each experiment. Quantitative analysis of pixel intensity (ESCRT III components) was performed after normalizing to an unstained area. The pMLKL + cells were counted manually from 3 randomly chosen ROIs/well for each treatment condition along with DAPI + cells in the same ROIs to obtain the total percent of pMLKL + glutamatergic neurons. For assessing neurite degeneration, NfH-200 + beads were manually counted from 3 randomly chosen ROIs of 200µm^2^ area/well for each treatment condition and reported as the average number of beads/200 µm^2^. Total of 3 replicates per treatment condition for all iPSC experiments.

### Statistical analysis

All human post-mortem data was assumed to be sampled from a non-Gaussian distribution and non-parametric analysis methods applied. The difference between two groups was compared using the unpaired Mann–Whitney test. Spearman correlation was used to test for associations between groups and the Spearman *r* and *p* values reported in each instance. One-way ANOVA with Bonferroni´s post-hoc correction was performed for multiple comparisons. Paired t-tests were used for comparing two groups in culture experiments. A two-sided *p*-value < 0.05 was considered significant.

## Results

### Necroptotic signaling is highly activated in AD hippocampus

**Fig. 1:**
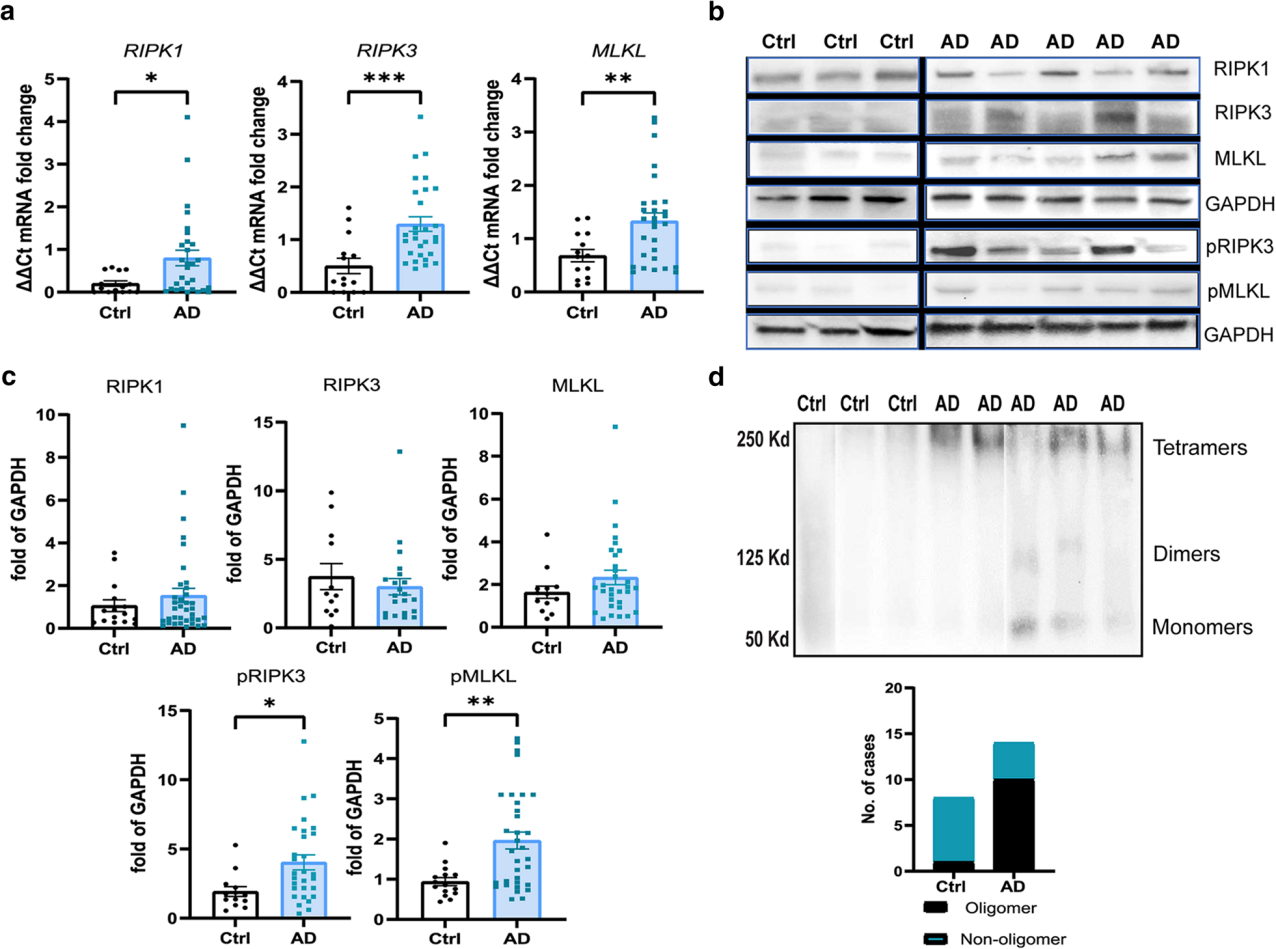
Activation of necroptosis signaling in AD hippocampus. **a** Analysis of mRNA levels of the RIPK1, RIPK3, and MLKL genes in the hippocampus grey matter in AD cases (n = 30) and controls (n = 14). **b** Representative western blots of RIPK1, RIPK3/pRIPK3, and MLKL/pMLKL. Full blots are shown in Additional file 6: Fig. 6. **c** Quantification of RIPK1, RIPK3, MLKL, pRIPK3, and pMLKL protein levels in AD (n = 20) and control cases (n = 10), normalized to GAPDH. **d** Western blot analysis of MLKL from hippocampal grey matter tissue lysate extracted in native conditions, showing the presence of oligomers. Quantification of the MLKL oligomers (~ 250 kDa) in the AD (n = 14) and control (n = 8) cases. For two group comparisons, the Mann–Whitney test was used. Data are represented as mean ± SEM, *p < 0.05, **p < 0.01, ***p < 0.001, ****p < 0.0001

**Fig. 2:**
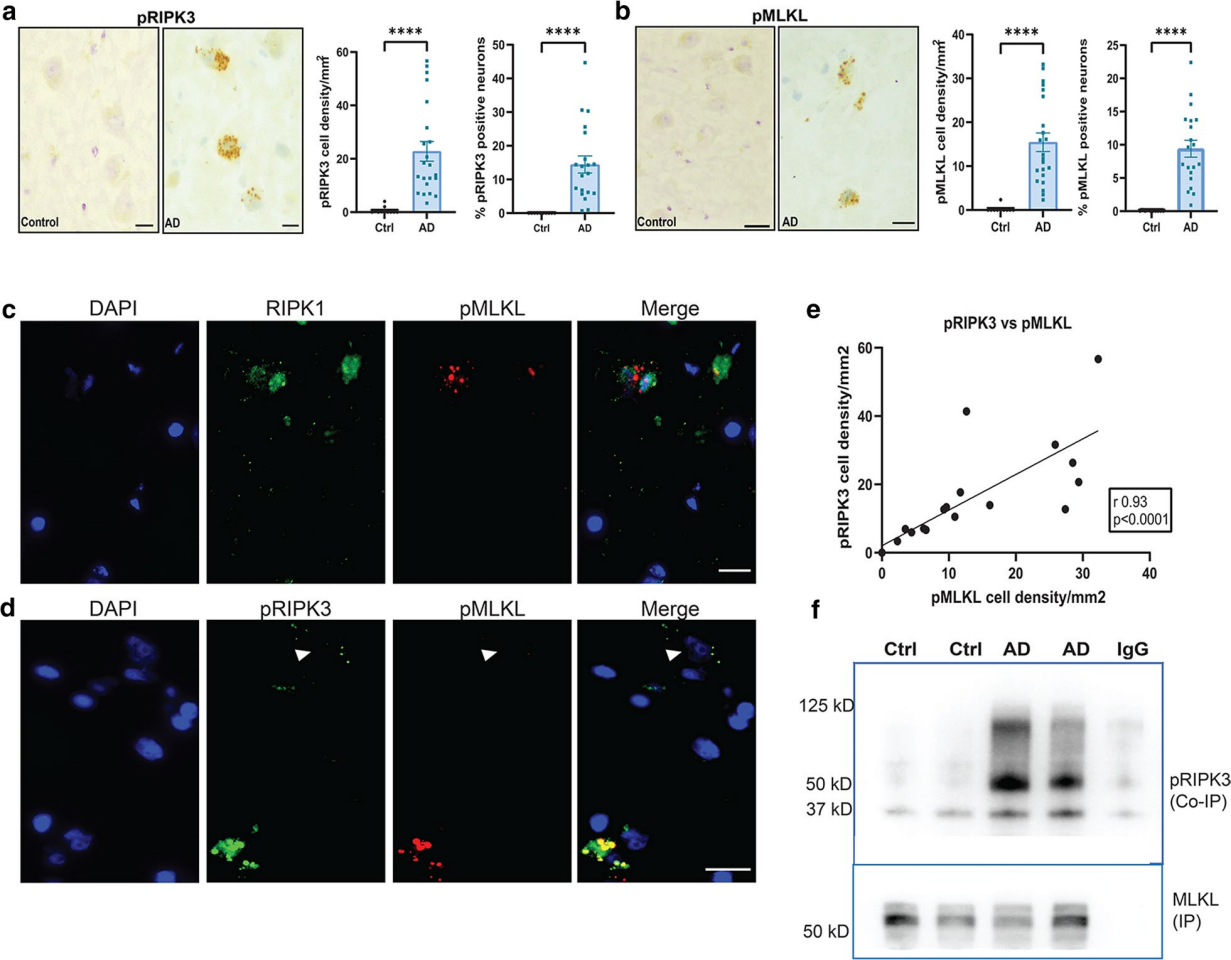
Necrosome formation in the AD hippocampus. **a** Representative images of pRIPK3 IHC in the hippocampal CA1 region from a control and AD case (scale bar = 20 µm) and the quantification of pRIPK3 + cell density and percentage of pRIPK3 + neurons in AD (n = 22) and control (n = 10) cases. **b** Representative images of pMLKL IHC in the hippocampal CA1 region from a control and AD case (scale bar = 20 µm) and the quantification of pMLKL + cell density and percentage of pMLKL + neurons in AD (n = 22) and control (n = 10) cases. Data are represented as mean ± SEM, ****p < 0.0001. **c** Representative immunofluorescence image of RIPK1 (green) and pMLKL (red) with DAPI + cell nuclei (blue) in CA1 pyramidal neurons of an AD case (scale bar = 20 µm). **d** Representative immunofluorescence image of pRIPK3 (green) and pMLKL (red) with DAPI + cell nuclei (blue) in CA1 pyramidal neurons of an AD case (scale bar = 20 µm). Some neurons are pRIPK3 + , but pMLKL- (arrowhead). **e** Correlation analysis between pRIPK3 and pMLKL cell densities in AD cases (*r* = 0.93, *p* < 0.0001). **f** Co-immunoprecipitation analysis of pRIPK3 (Co-IP) with MLKL (IP) in the hippocampal grey matter lysates from 2 AD and 2 control cases. Correlation analysis by Spearman comparison

In order to demonstrate the activation of necroptotic signaling in hippocampal neurons in the AD brain, we studied the detailed changes in this pathway at both mRNA and protein levels in brain tissues from 20 to 30 AD patients and 10 non-neurological controls. There was a two–threefold increase in the mRNA expression levels for *RIPK1* (mean ± SEM AD: 0.8 ± 0.18, Ctrl: 0.2 ± 0.06; *p* = 0.0152), *RIPK3* (AD: 1.3 ± 0.14, Ctrl: 0.5 ± 0.15; *p* = 0.0007) and *MLKL* (AD: 1.33 ± 0.15, Ctrl: 0.68 ± 0.12; *p* = 0.0057) in AD cases (Fig. 1a). Although there were some differences in the levels of RIPK1, RIPK3, and MLKL protein in the UREA soluble fraction, determined by western blotting, these were not significantly different between controls and AD, mainly due to the high degree of heterogeneity in the AD cases (Fig. 1b, c). In contrast, the activated pRIPK3 protein (AD: 4.04 ± 0.54; Ctrl: 1.93 ± 0.36; *p* = 0.0101) and pMLKL protein (AD: 1.96 ± 0.21; Ctrl: 0.94 ± 0.1; *p* = 0.0024) levels were significantly upregulated in the hippocampal grey matter of AD cases compared to controls (Fig. 1b, c). Using non-reducing conditions to probe the oligomeric state of MLKL, we found the almost exclusive presence of MLKL oligomers of approximately 250 kDa in the AD hippocampus, most likely representing tetramers (Fig. 1d). Immunohistochemical (IHC) localization of the expression of pRIPK3 and pMLKL, demonstrated pMLKL + (Fig. 2a) and pRIPK3 + (Fig. 2b) staining in the AD hippocampus, but not in the controls. Although predominantly found in the CA1-2 neurons in our cohort, 4 AD cases also showed a few pMLKL + neurons in the CA3 region, suggesting that neurons beyond CA1-2 regions can also show activation of necroptosis. The staining was characteristic of the granulovacuolar degeneration (GVD) bodies in pyramidal neurons and 9.4% ± 1.3% and 14.45% ± 2.55% of total neurons in the CA1-2 regions of AD cases expressed pMLKL and pRIPK3 respectively, compared to 0.04 ± 0.03% and 0.003% ± 0.002%, in controls (Fig. 2a, b; Additional file 1: Figure 1a). There was no colocalization of pMLKL immunostaining with astrocytes or microglia (Additional file 1: Fig. 1b). In addition, we also observed significantly higher numbers of pMLKL (twofold) and pRIPK3 (2.5-fold) positive neurons in female AD brains when compared to male AD brains (Additional file 1: Figure 1c), together with a significant increase in pRIPK3 protein levels and a trend towards increased pMLKL levels in the female AD brain (Additional file 1: Figure 1d). Therefore, our data suggests activation of the necroptosis pathway in CA1-2 hippocampal pyramidal neurons in AD brain with evidence of sexual dimorphism.

### Necrosome formation in the AD hippocampus

Previous studies have shown that the necrosome complex is formed when RIPK1 binds to and phosphorylates RIPK3, which further binds to and phosphorylates MLKL, leading to the oligomerization and migration of MLKL to the plasma membrane [65]. In this regard, the presence of pMLKL is an indication of activation of the necroptosis pathway. Therefore, we have considered this a marker for necroptosis throughout the study. We observed co-expression of pMLKL along with RIPK1 (Fig. 2c) and pRIPK3 (Fig. 2d) in the same neurons in the AD hippocampus. Interestingly, all pMLKL + neurons were also pRIPK3 + , showing colocalization at the subcellular level (Fig. 2d), but some pRIPK3 + pMLKL- neurons (arrowhead) were also present. This suggests that these neurons are at different stages of the necroptosis pathway. We also found a strong correlation between pRIPK3 + and pMLKL + cell densities in the AD cases (Fig. 2e). Co-immunoprecipitation of pRIPK3 with MLKL could be demonstrated in the AD hippocampus but not in the controls (Fig. 2f), which further provides evidence for the activation of the necroptosis pathway and formation of the necrosome complex in the AD hippocampus.

### Association between pMLKL + neurons and AD pathology

**Fig. 3:**
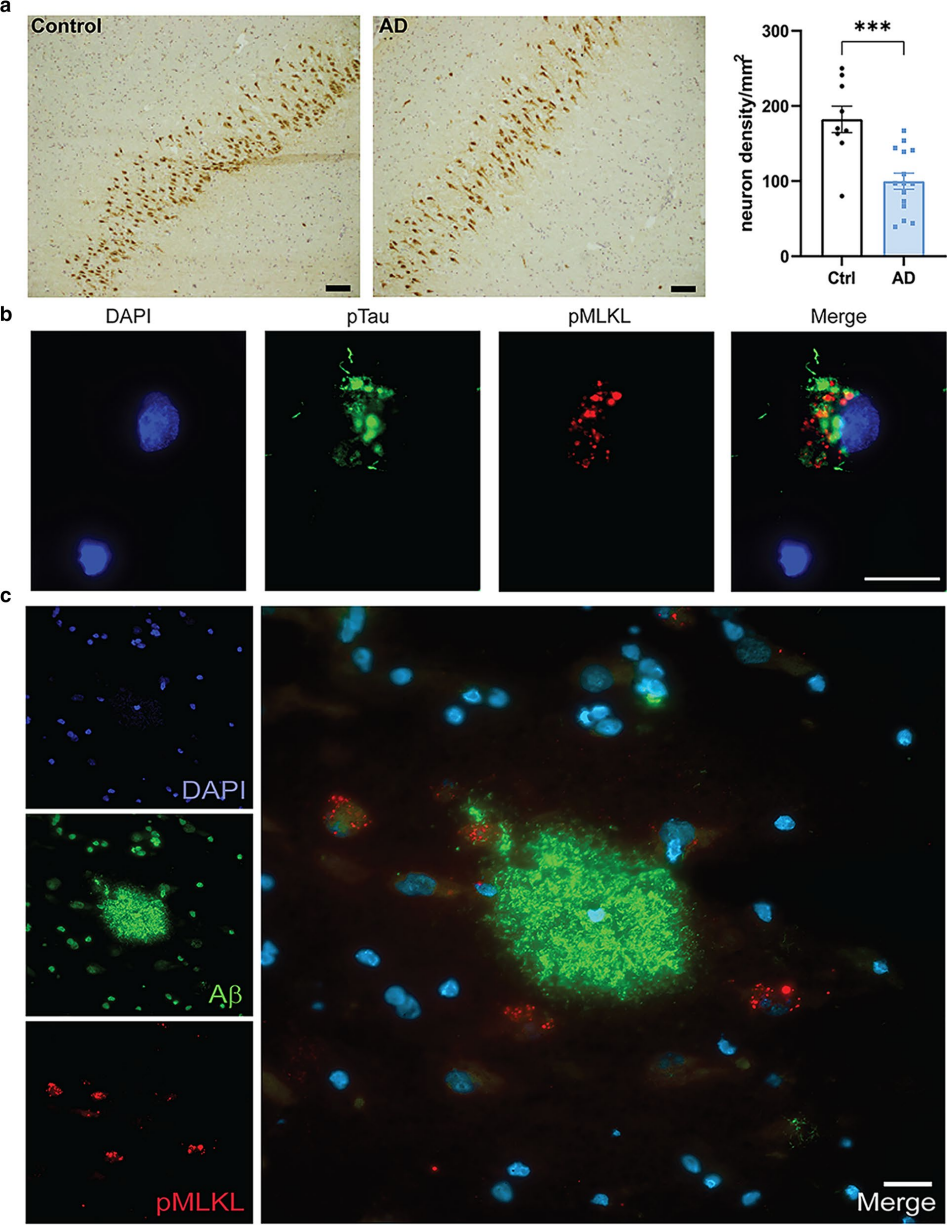
Association of pMLKL with AD pathology. **a** Representative images of CA1 pyramidal neurons stained with HuC/HuD antibody in the control and AD brain sections (scale bar = 100 µm); Quantification of the neuron density per mm^2^ in the control (n = 9) and AD (n = 15) hippocampal CA1 region. Data are represented as mean ± SEM, ***p < 0.001. **b** Representative immunofluorescence image of pTau (green) and pMLKL (red) with DAPI + cell nuclei in CA1 region of AD hippocampus. **c** Representative immunofluorescence image of Aβ (green) and pMLKL (red) with DAPI + cell nuclei in CA1 region of AD hippocampus. Scale bar = 20 µm

To investigate if there was any difference in neuron numbers between AD cases and control cases, we stained the hippocampal sections for the neuronal marker, HuC/HuD and calculated the neuron density per mm^2^ in the CA1-2 area. We observed that there was a significant 45% neuron loss in the AD hippocampus (99.95 ± 10.78 neurons/mm^2^) when compared with the control samples (182.3 ± 17.6 neurons/mm^2^; *p* = 0.0006) (Fig. 3a). In addition, we observed a strong negative correlation between total neuron density and pMLKL + cell density (*r* -0.73, *p* < 0.0001) and between total neuron density and pRIPK3 + cell density (*r* -0.63, *p* = 0.0014) (Additional file 1: Fig. 1e). A previous study on necroptosis in AD had suggested an association between necroptosis and Tau pathology, but not Aβ pathology [30]. In our AD cohort, we found that there was a positive correlation between pMLKL cell density and pTau levels, but not with Aβ levels (Additional file 2: Fig. 2a, b). Similarly, we found a significant positive correlation between pRIPK3 and pTau levels, but not between pRIPK3 and Aβ levels (Additional file 2: Figure 2e, f). pTau Braak stages and CERAD scores showed significant correlations with pMLKL + cell density (Additional file 2: Fig. 2c, d), but not with pRIPK3 cell density (Additional file 2: Fig. 2 g, h). Immunostaining showed co-expression of pTau + neurofibrillary tangles and pMLKL in a subset of neurons (Fig. 3b). In contrast, there was no overlap in the expression of Aβ and pMLKL within the neurons (Fig. 3c). However, pMLKL + neurons were often found in close proximity to Aβ plaques (Fig. 3c).

**Fig. 4:**
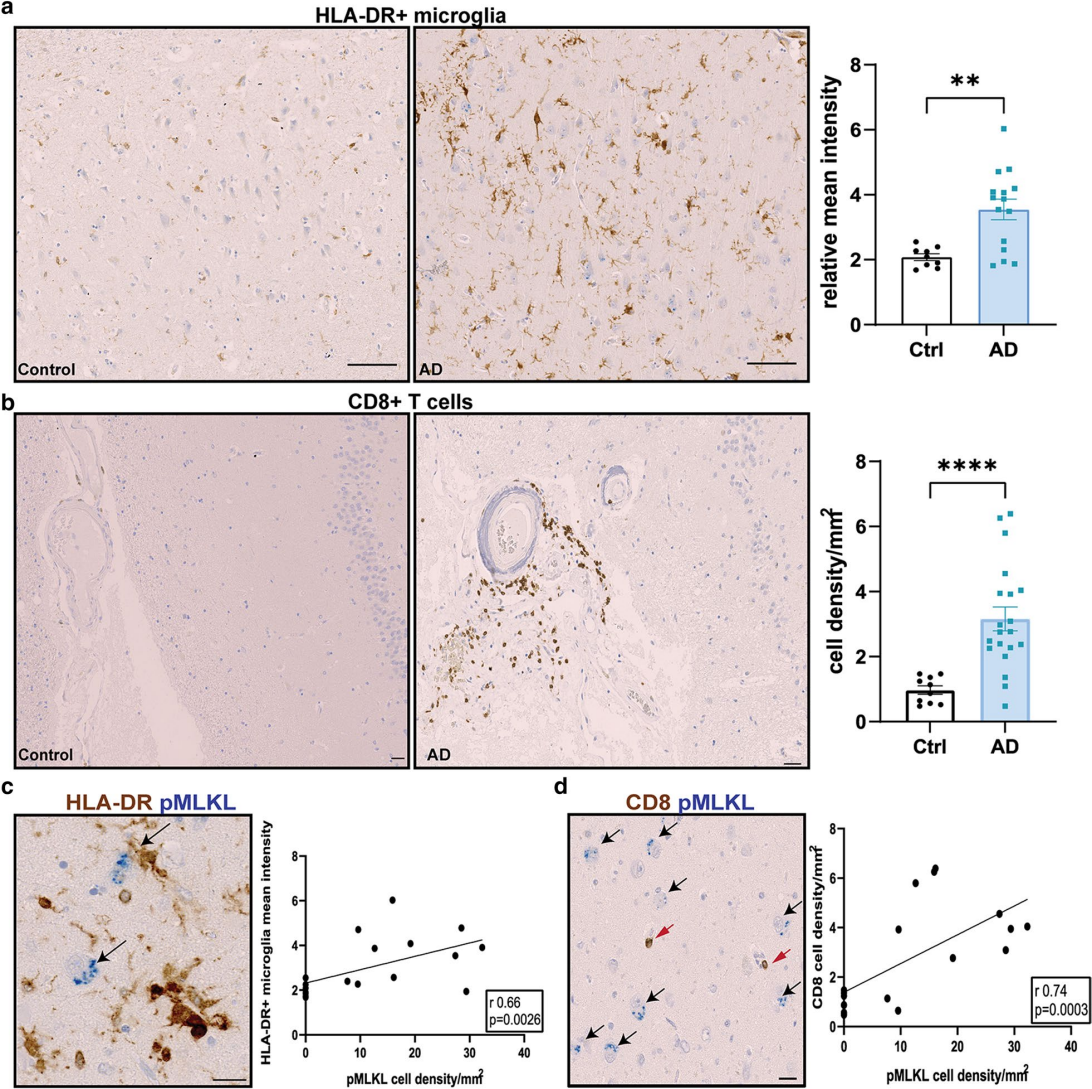
Association of pMLKL with inflammatory markers in AD. **a** Representative IHC and quantification of HLA-DR positive microglia in control (n = 9) and AD (n = 15) brain sections (scale bar = 100 µm). Data are represented as mean ± SEM, **p < 0.01. **b** Representative IHC showing CD8 + T cells (brown) in the control (left, n = 9) versus AD (right, n = 20) tissues (scale bar = 100 µm). **c** Representative image showing pMLKL + neurons (black arrows) in close proximity to HLA-DR + microglia (scale bar = 20 µm), along with correlation analysis between mean HLA-DR intensity and pMLKL + neuron density (*r* = 0.66, *p* = 0.0026). **d** Representative image showing pMLKL + neurons (black arrows) in close proximity to CD8 + T cells (red arrows) (scale bar = 20 µm), along with correlation analysis between CD8 + T cell density and pMLKL + neuron density (*r* = 0.74, *p* = 0.0003). Correlation analysis by Spearman comparison

The AD brain sections showed a significantly increased presence of HLA-DR + microglia compared to the control sections, as measured by the mean HLA-DR intensity (Ctrl: 2.08 ± 0.1, AD: 3.55 ± 0.32; *p* = 0.0035), suggesting significant microglial activation in the AD tissue (Fig. 4a). The activated HLA-DR + microglia in the AD brains were present both in the white matter and grey matter, with morphologies ranging from moderately hypertrophic cell bodies to highly activated enlarged cell bodies, similar to those reported in AD previously [66]. We also observed increased numbers of CD8 + T cells in the AD cases compared to the control cases, which were predominantly present in the perivascular spaces and meninges, but were also observed to a lesser extent within the parenchyma, especially in the AD hippocampus (Fig. 4b). In the AD cases, the activated HLA-DR + microglia and the CD8 + T cells were observed in close proximity to pMLKL + neurons (Fig. 4c, d). Moreover, the mean intensity of HLA-DR staining and the overall CD8 + T cell density correlated significantly with the pMLKL + neuron density in the AD hippocampus (Fig. 4c, d). Thus, our data suggests several factors that could contribute to a chronic inflammatory microenvironment around specific CA1 neurons, increasing their susceptibility to necroptosis.

### TNFR1/RIPK1 signaling is upregulated in neurons in the AD hippocampus

**Fig. 5:**
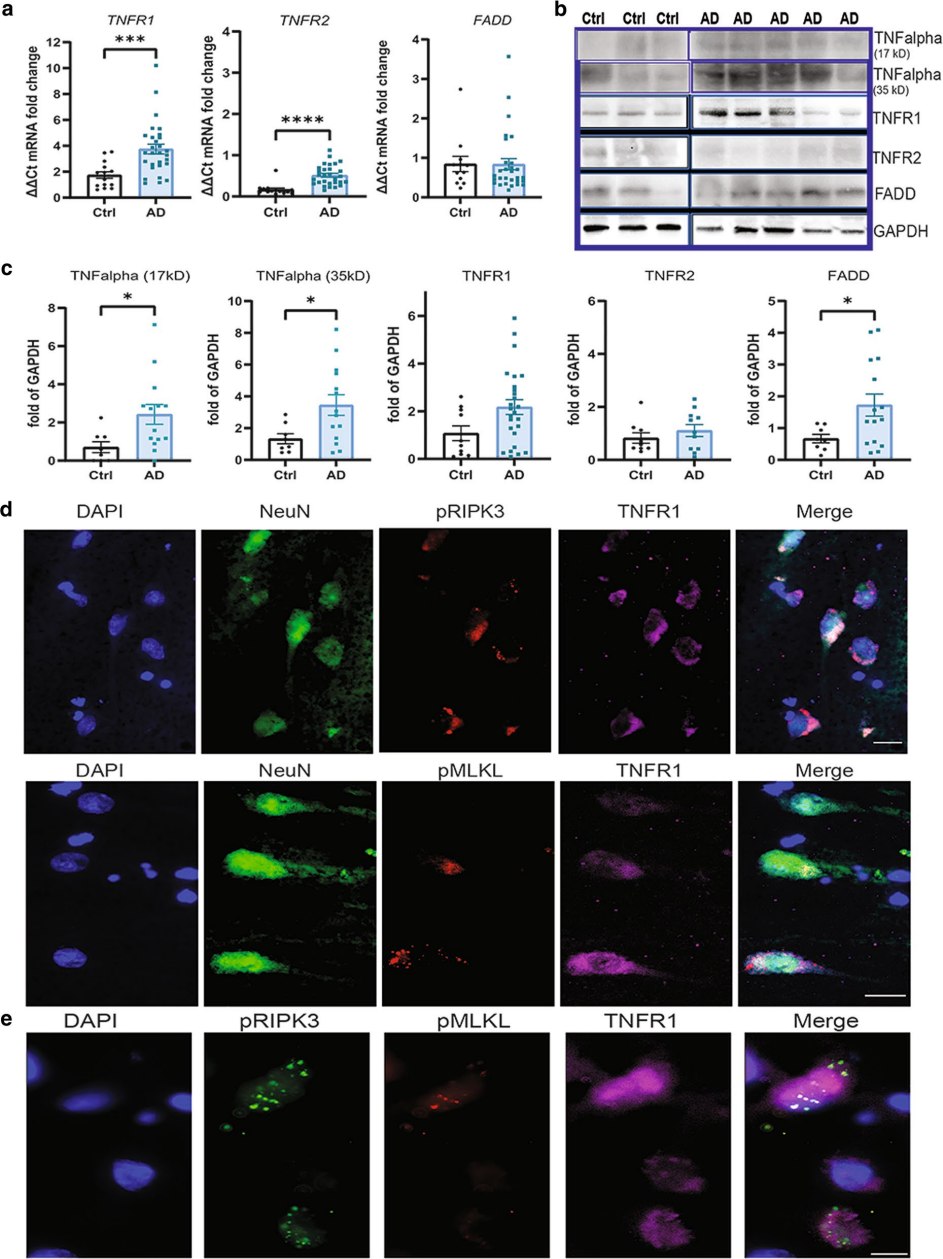
TNFR1-mediated necroptosis signaling is activated in the CA1 neurons in AD hippocampus. **a** Analysis of mRNA levels of the TNFR1, TNFR2, and FADD genes in the hippocampus grey matter in AD cases (n = 30) and controls (n = 14). **b, c** Representative western blots of the soluble and membrane bound forms of TNF and TNFR1, TNFR2, and FADD (full blots are shown in Additional file 6: Fig. 6) and their quantification in AD (n = 15) and control cases (n = 9), normalized to GAPDH. Data are represented as mean ± SEM, *p < 0.05, ***p < 0.001. **d** Representative immunofluorescence image of pRIPK3 (red, top panel) and pMLKL (red, bottom panel) and TNFR1 (pink) in NeuN + (green) CA1 neurons with DAPI + cell nuclei (blue) in an AD case (scale bar = 20 µm). **e)** Representative immunofluorescence image of pRIPK3 (green), pMLKL (red) and TNFR1 (pink) with DAPI + cell nuclei (blue) in an AD case (scale bar = 20 µm)

The initiating factors underlying necroptotic cell death in neurodegenerative diseases are still unclear. In non-neuronal model systems, TNF is the best-known trigger of necroptosis [76]. We have previously demonstrated the presence of TNF-mediated necroptosis signaling in MS [46]. Therefore, we investigated the link between TNF signaling and necroptosis in the AD brain by determining the expression levels of TNF receptors 1 and 2, and a key initial regulator Fas associated death receptor (FADD). A significant upregulation of *TNFR1* (Ctrl: 1.74 ± 0.25, AD:3.75 ± 0.38; *p* = 0.0002) and *TNFR2* (Ctrl:0.17 ± 0.04, AD: 0.51 ± 0.05; *p* < 0.0001) gene expression, but no change in *FADD* gene expression, was observed in the AD brain compared to controls (Fig. 5a). Western blot analysis showed a significant increase in both soluble TNF (17 kDa; Ctrl: 0.7 ± 0.23, AD:2.42 ± 0.52; *p* = 0.0247), the membrane-bound form of TNF (35 kDa; Ctrl: 1.33 ± 0.31, AD: 3.46 ± 0.65; *p* = 0.0421) and FADD (Ctrl: 0.67 ± 0.13, AD: 1.73 ± 0.35, *p* = 0.0473) levels. There was a clear trend towards an increase in TNFR1 protein levels (Ctrl: 1.08 ± 0.31, AD: 2.18 ± 0.31; *p* = 0.063), which did not reach significance due to substantial heterogeneity between cases, but no difference in TNFR2 levels (Fig. 5b, c). Interestingly, we also observed a trend towards increased TNFR1 and TNF levels in the female AD brain samples as compared to the male AD brain samples (Additional file 1: Figure 1d). As reported previously [71], we observed a low level of TNFR1 expression in all CA1 neurons in both control and AD cases. However, triple immunofluorescence revealed increased TNFR1 expression specifically in neurons in the AD hippocampus that were pRIPK3 + or pMLKL + (Fig. 5d, e). Together with the demonstration of upregulation and localisation of the downstream components of the necroptosis pathway (Figs. 1, 2), these results strongly suggest a TNFR1-mediated activation of necroptosis in these neurons in the AD hippocampus.

### Apoptosis signaling is not activated in the AD hippocampus

**Fig. 6:**
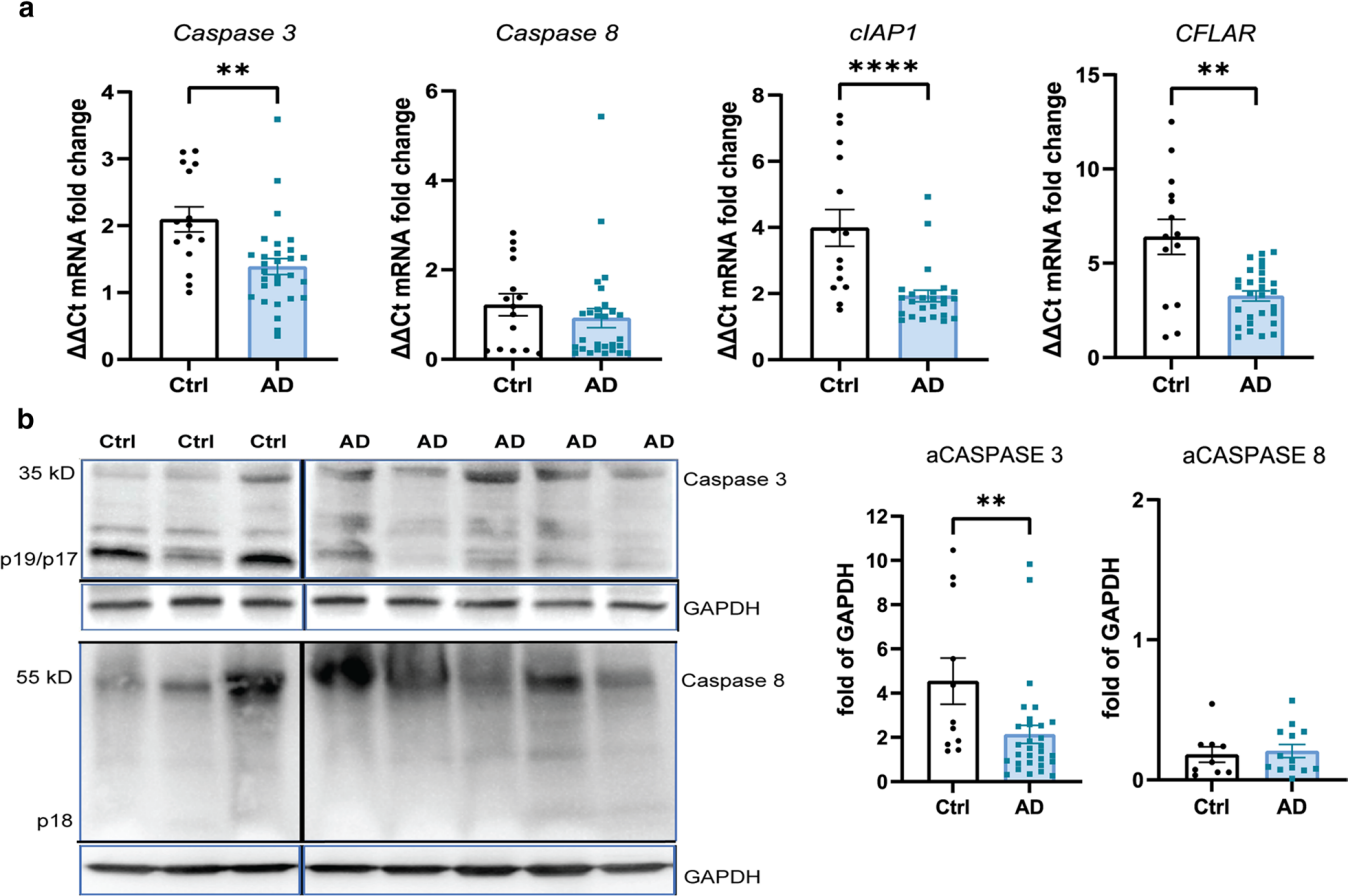
Apoptosis signaling is downregulated in AD hippocampus. **a** Analysis of mRNA levels of the Caspase 3, Caspase 8, cIAP1, and CFLAR genes in the hippocampus grey matter in AD cases (n = 30) and controls (n = 14).** b** Representative western blots of full-length and cleaved caspase 3 and caspase 8. Full blots are shown in Additional file 6: Fig. 6. Quantification of cleaved caspase 3 and caspase 8 protein levels in AD (n = 15) and control cases (n = 10), normalized to GAPDH. Data are represented as mean ± SEM, **p < 0.01, ***p < 0.001, ****p < 0.0001

To determine the extent of apoptosis signaling in the AD hippocampus, we next studied the activation of caspase dependent apoptosis. RT-PCR analysis showed a significant decrease in *CASP3, cIAP1* and *cFLAR* expression levels in the AD brain (Fig. 6a). Caspase-dependent apoptosis is initiated by proteolytic cleavage of caspase-8 (55 kDa) into the p18 and p43 subunits [22]. The protein levels of the cleaved active p18 subunit were not significantly different from that in control samples (Fig. 6b). However, the downstream cleaved caspase-3 levels were significantly lower in the AD brain when compared to control samples (Fig. 6b). These results, along with lower gene expression levels of *cFLAR* and *cIAP1*, indicate that caspase-dependent apoptosis is not significantly activated in neurons in AD, which suggests that extrinsic activation of the apoptotic pathway is not the main mechanism driving cell death in neurons in AD.

### ESCRT III pathway proteins are altered in AD hippocampus

**Fig. 7:**
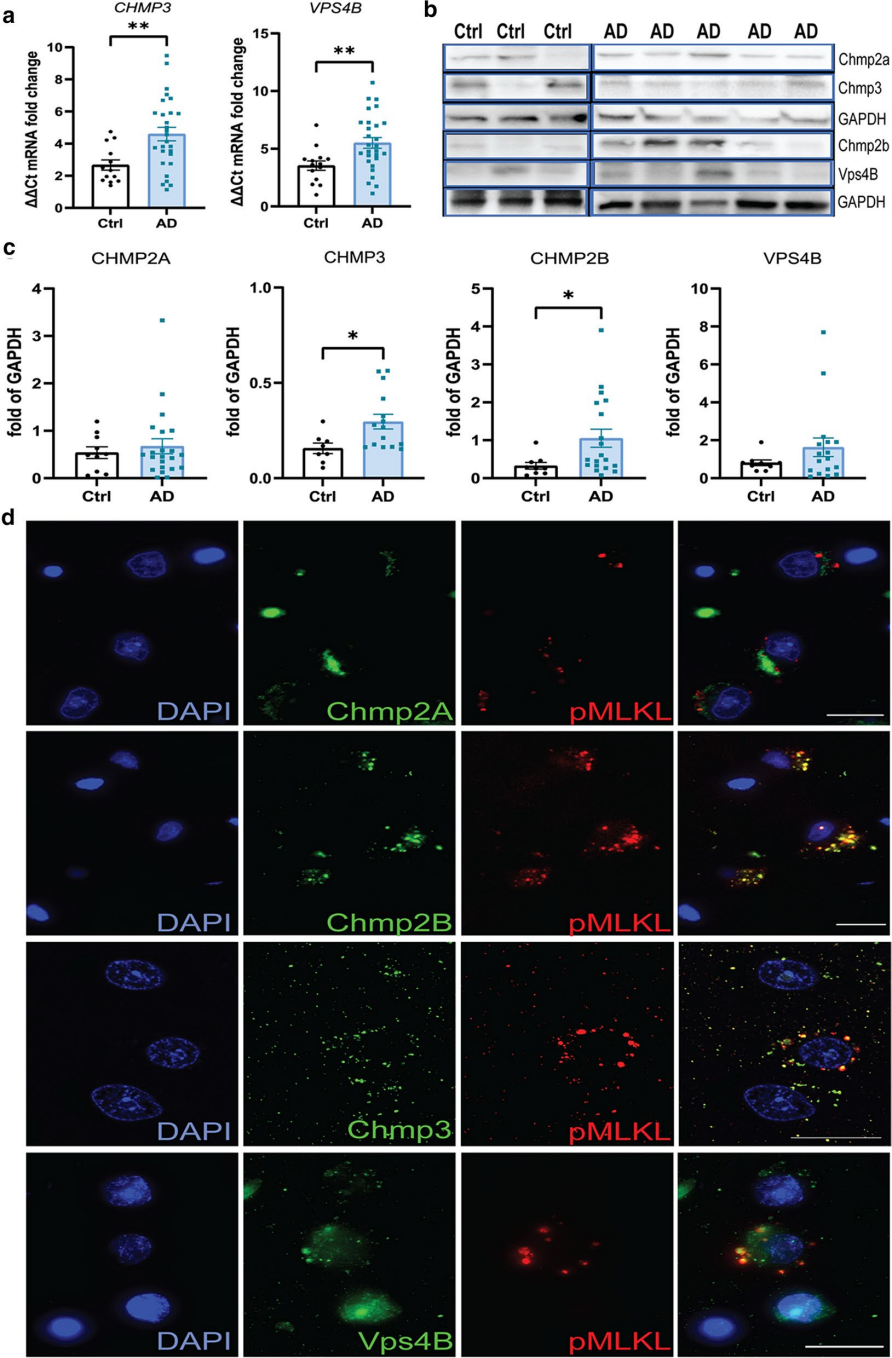
Altered expression of ESCRT III components in the AD hippocampus. **a** Analysis of mRNA levels of the Vps24, and Vps4b genes in the hippocampus grey matter in AD cases (n = 30) and controls (n = 14).** b** Representative western blots of CHMP2A, VPS24, CHMP2B, and VPS4B. Full blots are shown in Additional file 6: Fig. 6. **c** Quantification of CHMP2A, VPS24, CHMP2B, and VPS4B protein levels in AD (n = 20) and control cases (n = 10), normalized to GAPDH. Data are represented as mean ± SEM, **p < 0.01. **d** Representative immunofluorescence image of Chmp2A (green, top row), Chmp2B (green, second row), Vps24 (green, third row), and Vps4B (green, bottom row) with pMLKL (red) and DAPI + cell nuclei (blue) in the CA1 neurons of AD cases (scale bar = 20 µm)

The endosomal sorting complexes required for transport group III (ESCRT III) proteins, have been recently implicated as a counterbalance for necroptosis and a possible inherent mechanism to prevent or delay cell death after necroptosis has been triggered. Conversely, silencing of ESCRT-III can induce necroptosis in cells [16]. CHMP2B, a component of the ESCRT III complex, is also a marker for GVD bodies in the AD brain [69]. Therefore, we investigated if key components of the ESCRT-III pathway showed altered expression in the AD hippocampus. Gene expression analysis of the essential components of the ESCRT III complex (*CHMP2a, CHMP2b, VPS24/CHMP3, CHMP4b and CHMP6)* and *VPS4b,* which is important for recycling the ESCRT-III components, showed that *VPS4b* and *CHMP3* had significantly higher expression in the AD brains compared to the controls (Fig. 7a, Additional file 3: Figure 3a). At the protein level, however, only Chmp3 showed a significant increase in the AD brain (Ctrl: 0.16 ± 0.03, AD: 0.3 ± 0.04; *p* = 0.0282) (Fig. 7b, c). Since CHMP2B is a GVD marker and implicated in AD, we also investigated CHMP2B protein levels along with its partner CHMP2A, although we did not observe significant difference at the mRNA level. CHMP2B protein showed a significant increase in the AD samples (Fig. 7b. c; Ctrl: 0.33 ± 0.09, AD:1.05 ± 0.24; *p* = 0.0215), whereas no difference was observed for CHMP2A. Co-expression of ESCRT-III components was present in pMLKL + neurons, with a significant overlap at the subcellular level for CHMP2B, CHMP3 and VPS4B, confirming pMLKL localization in the GVD bodies (Fig. 7d). Our data suggest that the activation of necroptosis might alter the expression levels of ESCRT III proteins in AD as a compensatory mechanism.

### TNF induces necroptosis activation in iPSC derived human glutamatergic neurons

**Fig. 8:**
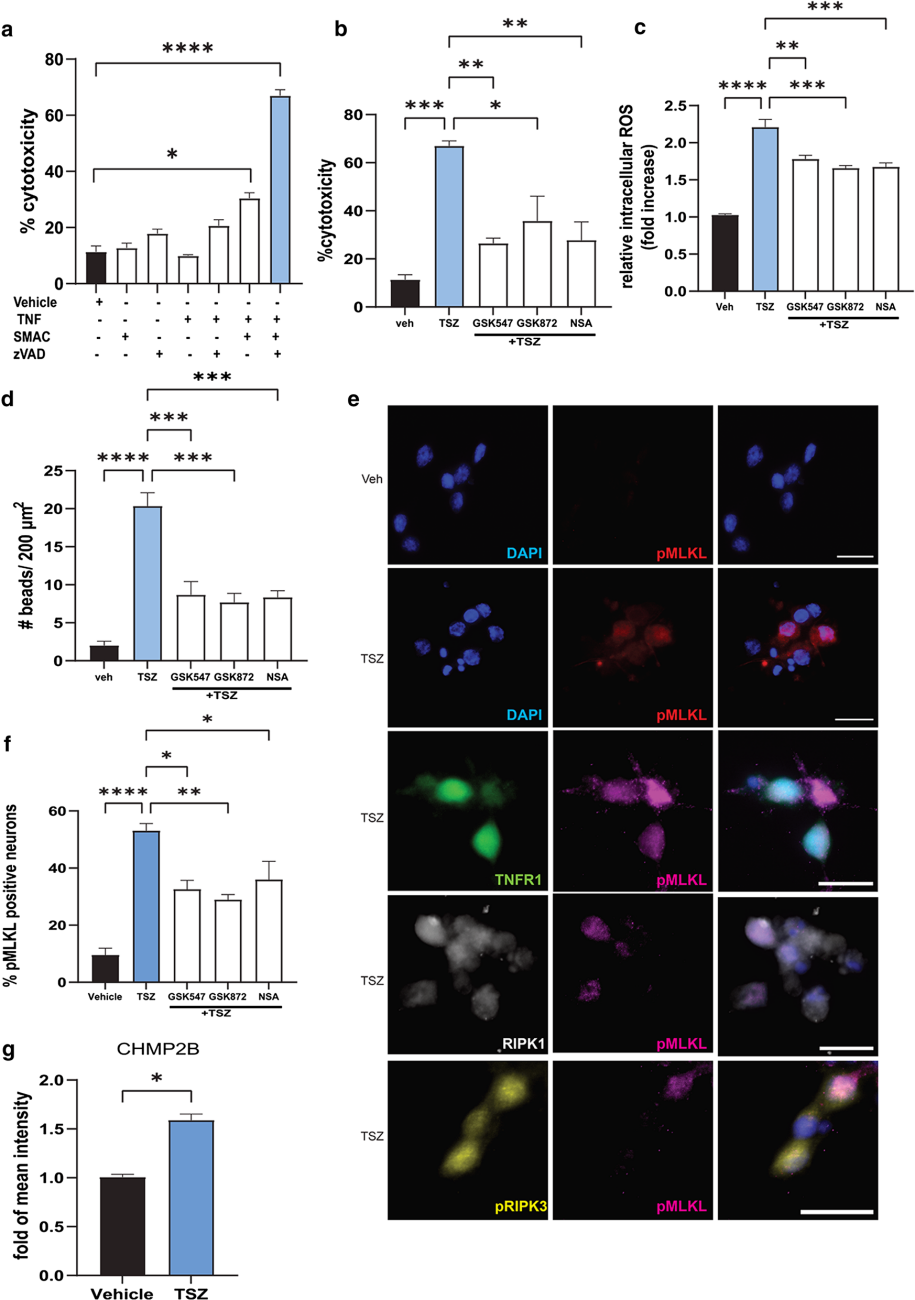
TNF mediated activation of necroptosis in human iPSC-derived glutamatergic neurons when apoptosis is inhibited. **a** Viability of human iPSC-derived glutamatergic neurons treated with combinations of TNF (100 ng/mL), SMAC mimetic (2 µM), and zVAD (10 µM) [TSZ], or vehicle control, assessed by LDH assay at 24 h. **b** Viability of human iPSC-derived glutamatergic neurons treated with vehicle control, TSZ, or TSZ with GSK-547 (10 nM), GSK-872 (100 nM), or necrosulfonamide (NSA, 1 µM), assessed by LDH assay at 24 h. **c** Quantification of intracellular ROS in human iPSC-derived glutamatergic neurons treated with vehicle control, TSZ, or TSZ with GSK-547, GSK-872, or NSA. **d** Quantification of neurite beading in human iPSC-derived glutamatergic neurons treated with vehicle control, TSZ, or TSZ with GSK-547, GSK-872, or NSA. **e** Representative images of human iPSC-derived glutamatergic neurons treated with vehicle or TSZ immunostained for pMLKL (red) and DAPI (blue), along with representative images of TSZ treated neurons co-stained for TNFR1 (green) and pMLKL (pink), RIPK1 (white) and pMLKL (pink), and pRIPK3 (yellow) and pMLKL (pink) (scale bar = 20 µm). **f** Quantification of pMLKL + neurons treated with vehicle control, TSZ, or TSZ with GSK-547, GSK-872, or NSA. **g** Quantification of Chmp2b mean intensity normalized with DAPI mean intensity. All quantification performed with 3 replicates per group per experiment. One-way ANOVA with Bonferroni’s post-hoc correction for multiple group comparisons, and t-test for comparing between two groups. Data are represented as mean ± SEM, *p < 0.05, **p < 0.01, ***p < 0.001, ****p < 0.0001

In order to confirm that TNF can indeed induce necroptosis in human neurons, human iPSC-derived glutamatergic neurons were treated under different conditions for 24 h at day 9–11 post-plating and assessed for cell cytotoxicity by measuring the release of lactate dehydrogenase (LDH) into the culture medium. Before starting the treatments, we ascertained that the neurons exhibited neurite outgrowth and confirmed their homogenous expression of pan-neuronal protein MAP2 and glutamatergic neuron-specific transporter, VGLUT1 (Additional file 4: Fig. 4a). TNF alone (100 ng/ml) was not sufficient to induce neuronal cell death, nor were the pan-caspase inhibitor z-Val-Ala-Asp (Ome)-fluoromethylketone (z-VAD; 10 μM) or a SMAC mimetic (SMAC; 2 μM), when added alone (Fig. 8a). TNF and SMAC mimetic together increased LDH release significantly compared to vehicle treatment, which was further increased in a time-dependent manner when all three were combined together (TSZ treatment), reaching 67% cytotoxicity after 24 h treatment (Fig. 8a, Additional file 4: Fig. 4b). To further define the role of necroptosis in cell death induced by TSZ exposure, we pharmacologically inhibited RIPK1, RIPK3, or MLKL with GSK547 (10 nM), GSK872 (100 nM), or necrosulfonamide (NSA, 1 µM), respectively, and demonstrated that all the inhibitors individually significantly reduced the cytotoxicity by 30–55% (Fig. 8b). Increased reactive oxygen species (ROS) production by mitochondria has also been shown to promote RIP autophosphorylation and is required for RIP3 recruitment into the necrosome [74]. Therefore, we determined the levels of intracellular ROS production following TSZ treatment of human neurons, with or without the RIPK1, RIPK3 and MLKL inhibitors. We found that there was a significant increase (> twofold) in ROS production with TSZ treatment even by 6 h post-treatment, which was effectively reduced by approximately 25% in the presence of the inhibitors (Fig. 8c, Additional file 4: Fig. 4c).

Immunostaining with neurofilament-H (NfH)-200 antibody showed axonal/neurite beading at 8 h post-TSZ treatment, which further increased at 16 h post-treatment (Additional file 4: Fig. 4d) when compared to vehicle treated neurons. Quantification of the number of beads across different treatment conditions at 16 h of TSZ treatment showed a tenfold increase compared to vehicle treatment, indicative of neurodegeneration. Treatment with RIPK1, RIPK3 or MLKL inhibitors decreased this number significantly compared to TSZ treatment alone (Fig. 8d, Additional file 4: Fig. 4d). Next, we investigated the expression of pMLKL as an indicator of activation of necroptosis across different treatment conditions at 16 h post-treatment. Upregulation of pMLKL expression was observed in the cytoplasm of the treated neurons in response to TSZ (Fig. 8e, f). Moreover, pMLKL + neurons were also positive for TNFR1, RIPK1, and pRIPK3 expression (Fig. 8e). Pre-treatment with RIPK1, RIPK3 and MLKL inhibitors reduced the number of pMLKL positive neurons (Fig. 8f).

Since some of the ESCRT III components were altered in the AD brain, we also investigated their expression levels following TSZ treatment in the iPSC-derived glutamatergic neurons. Chmp2B showed a significantly increased expression with TSZ treatment (Fig. 8g, Additional file 5: Figure 5a), with no significant changes observed for Chmp2A, Chmp3, and Vps4B (Additional file 5: Fig. 5c). Neurons expressing higher levels of Chmp2B had lower pMLKL expression (arrows) and vice versa (arrowhead) (Additional file 5: Figure 5b). Taken together, these results demonstrate that TNF can trigger necroptotic cell death in human glutamatergic neurons through MLKL activation, but only after the alternative TNF-signaling pathways have been inhibited. Small molecule inhibitors of RIPK1, RIPK3, and MLKL were able to significantly reduce cytotoxicity, ROS production and neurite beading in the TSZ treated neurons. In addition, TSZ treatment resulted in increased Chmp2B expression in these neurons, possibly to counteract the increase in pMLKL in those neurons.

## Discussion

Recently, we demonstrated that a local increase in TNFR1 expression on the cortical grey matter neurons in progressive MS can be linked to necroptosis signaling and neurodegeneration, in the presence of reduced apoptotic signaling [46]. Because of the extensive interest in inflammatory mechanisms in neurodegeneration in other neurological disorders [11, 15, 21, 32, 59, 72], we extended our study to the AD brain. Here, we demonstrate a link between TNF signaling via TNFR1 and necroptosis activation and neuronal loss in the AD hippocampus using human post-mortem brain tissues. This was recapitulated in vitro in human iPSC-derived glutamatergic neurons treated with TNF when apoptosis was inhibited. In addition, our findings show that small molecule inhibitors against RIPK1, RIPK3, and MLKL could significantly protect human cortical neurons against TNF-mediated necroptotic cell death in vivo. We also demonstrate related changes in the ESCRT-III pathway components in the AD hippocampus. These findings provide new insights into the involvement of TNF signaling and necroptosis in neurodegeneration in AD.

Our results showing an increase in the expression of necroptosis pathway genes and proteins in the AD brain is consistent with previous studies on necroptosis activation [5, 30, 45], but build on this to show co-expression of RIPK1 and pRIPK3 with pMLKL in the same neurons, and coimmunoprecipitation of pRIPK3 with MLKL, indicating interaction and necrosome formation in these neurons. Upon phosphorylation, MLKL is suggested to oligomerize in the cytoplasm and migrate to the plasma membrane, where it causes membrane destabilization and ultimately cell death [65]. We also demonstrate the presence of MLKL oligomers, most likely dimers and tetramers, exclusively in the AD samples. Expression of the activated proteins involved in the final stages of necroptosis, pRIPK3 and pMLKL, was predominantly seen in the large pyramidal neurons of the CA1-2 regions of the hippocampus, which is the most vulnerable region for AD-related neurodegeneration, suggesting that specific neurons in these regions could be differentially vulnerable to the inflammatory microenvironment. Although we only studied the AD hippocampus, it is likely that necroptosis would also be responsible for neuronal loss in neocortical regions implicated in AD, as suggested by a previous study [30], but further studies are necessary to fully substantiate this.

Neurons expressing pTau tangles were often also pMLKL positive, suggesting a link between pTau and necroptotic cell death. pMLKL expression levels correlated significantly with pTau expression but not with Ab expression, consistent with a previous study [30]. However, pMLKL + neurons were frequently found in close proximity to Aβ plaques, which are known to produce and secrete several inflammatory factors including chemokines and cytokines such as TNF [36]. While determining other factors that could contribute to a chronic inflammatory microenvironment in the hippocampal region, we observed an increase in activated microglia, assessed by HLA-DR staining and hallmark morphological changes. Interestingly, the pMLKL + neurons were often observed in close proximity to these activated microglia with a strong overall correlation between the pMLKL + neuron density and HLA-DR + microglial staining. Together with the observation that there was an increase in CD8 + T cells in the perivascular and meningeal regions in the proximity of pMLKL + neurons in the hippocampus in some AD brains, this demonstrates the presence of neurons with activated necrosome proteins in a potentially inflammatory environment. Recent studies have shown that CD8 + T cells are not only found in the cerebrospinal fluid (CSF) in AD but can also contribute to the progression of cognitive impairment [15, 59], suggesting that these T cells could secrete cytotoxic and proinflammatory cytokines that affect the hippocampus. Since the hippocampus is surrounded by CSF, this creates the possibility for the CD8 + T cells to either infiltrate the hippocampus or for the neurons and microglia to be exposed to the secreted proinflammatory cytokines. Taken together, our data suggest that CA1-2 pyramidal neurons are susceptible to necroptosis under inflammatory conditions, consistent with the hypothesis that inflammation is one of the key drivers of neurodegeneration in AD.

Significantly elevated levels of necroptosis pathway proteins and increased numbers of degenerating neurons have been previously reported in 5xFAD mice, which are characterized by pronounced cell loss, when compared with non-transgenic littermates or with APP/PS1 mice that do not show significant cell loss [5, 45]. These effects were reduced in 5xFAD mice treated with the necroptosis inhibitor, necrostatin (Nec1S), compared to vehicle-treated animals, suggesting that necroptosis contributes to neuronal death in this AD model [5]. Consistent with these observations and previous data from human tissues [30], we observed a strong correlation between the increase in pMLKL + neuron density and the decrease in overall neuron density in the hippocampus. In addition, we also noted a similar negative relationship between pRIPK3 + neuron density and neuronal loss in the CA1 region. However, whether all neurons expressing these necroptosis activation markers will eventually die, or for how long the neurons express these markers before dying, cannot be ascertained from this data. The percentage of pRIPK3 + and pMLKL + neurons was relatively high (a mean of 14.5% and 9.5%, respectively) for a single snapshot in time, which suggests that necroptosis is likely to contribute to significant neuronal loss over the disease course. This is significantly higher than that observed previously for MS brains [46]. This difference may reflect the cumulative loss of neurons over a long disease duration in a slowly progressive disease like MS as compared to a more rapid neuron loss in a late-onset condition with a shorter disease duration like AD. Interestingly, we observed a significantly higher number of pRIPK3 + and pMLKL + neurons in the female AD brains compared to male AD brains. This was accompanied by a significantly higher pRIPK3 protein levels and a trend towards higher pMLKL, TNF, and TNFR1 protein levels in the female AD brain samples than in the male AD brain samples, although no significant sex difference was observed at the transcript level (data not shown). Taken along with our data showing a strong negative correlation between neuron density and pMLKL/pRIPK3 positive neurons, this could translate to increased neuron loss in the female AD brain than the male AD brain over the disease course. This supports previous findings that in patients with mild cognitive impairment and AD, brain volumes decline faster in women than in men [57], and that a negative correlation between Braak stages and neuronal density in hippocampus was observed in women, specifically in the CA1 region [35]. Our data suggests for the first time a sex difference in the activation of the necroptosis pathway that could make the female AD brain more susceptible to neuronal loss over the course of the disease and could contribute to gender being a main risk factor in AD. The role of estrogen in neuroprotection is well-established, and reduction of estrogen with age and after menopause have been associated with increased oxidative stress, mitochondrial dysfunction, neuroinflammation, and tau hyper-phosphorylation [63]. Studies have shown that older women with AD have lower brain levels of estrogens when compared to age-matched women without AD [50]. Thus, loss or reduced estrogen levels in women may contribute to increased risk for neuronal death in AD through several different pathways. In addition, other factors such as microglial miRNA which show sex-specific expression changes have been shown to influence microglial transcriptome and tau pathogenesis, thus perhaps influencing neuronal vulnerability in males and females with AD [29].

Apoptosis has long been debated to be responsible for neuronal loss in AD [51, 55], although previous post-mortem studies have shown morphological and molecular signs of apoptosis to be very rare, affecting only 0.02–0.1% of neurons [26]. In addition, some markers used to indicate apoptosis do not discriminate between necroptosis and apoptosis. It is unlikely that apoptosis is sufficient to explain the extent of neuronal loss in AD. In our cohort, apoptosis was not significantly activated in the AD brain compared to the control brain, supporting previous studies that show that caspase inhibition or insufficient caspase activation can lead to necroptosis [1, 13, 19]. Our observations showed that there was absence of apoptotic morphological features, and no caspase 8 activation in the AD and control brains. The absence of caspase 8 activation not only leaves the RIPK1 kinase domain intact to autophosphorylate and then phosphorylate downstream RIPK3 and MLKL [27, 65], but has also been shown to disturb the inflammatory homeostasis in mice [31]. Moreover, two Caspase 8 variants have been reported to be associated with AD in a large cohort study, both of which showed reduce functionality due to either significantly lower activity or sequestration [49]. Activated caspase 3 levels were lower in the AD hippocampus compared to the control hippocampus. In addition, at the mRNA level, caspase 3, and cIAP1 levels were decreased in the AD hippocampal tissue, suggesting reduced apoptosis in the AD tissues. The reduced *CFLAR* mRNA levels suggest decreased NF-κB activation, which would also increase necroptotic signaling in the absence of apoptosis [7, 56, 71]. Taken together with our data on the necroptosis pathway activation, these results point to necroptosis as a major form of cell death in the AD brain, although other forms of cell death, such as ferroptosis and pyroptosis cannot yet be excluded.

Although necroptosis activation has been recently demonstrated in AD [5, 30], the mechanism underlying the activation remains unclear. We propose that pro-inflammatory cytokines, such as TNF, can diffuse into the hippocampal grey matter from the CSF or be induced in glial cells by dying neurons/Aβ plaques/reactive microglia, to induce necroptosis and neurodegeneration via TNFR1 signaling, similar to our findings in MS cortical grey matter [6, 34, 46]. The increase in TNF levels in the hippocampal parenchyma and the co-expression of pRIPK3 and pMLKL in neurons with increased TNFR1 expression strongly suggests the activation of TNFR1-mediated signaling pathway in the hippocampal neurons in AD. Although TNFR1 signaling can trigger apoptosis, a decrease or no change in caspase-8 and caspase-3 cleavage, along with the increase in pRIPK3 and pMLKL levels, indicates a switch towards necroptosis rather than apoptosis in these neurons. In the presence of a caspase inhibitor and a SMAC mimetic, which extrinsically recapitulates our observations in the AD brain, TNF stimulation of iPSC derived human glutamatergic neurons led to the activation of necroptosis by increasing the pMLKL expression in the cytoplasm, which was reversed by RIPK1, RIPK3, or MLKL inhibitors. Interestingly, these inhibitors not only reduced the pMLKL expression in the treated neurons, but also largely restricted the pMLKL expression to the nucleus. This suggests that the presence of cytosolic pMLKL is important for necroptotic cell death, consistent with the finding that blocking nuclear export and increasing nuclear retention of pMLKL, reduces necroptotic cell death [68]. Using a rat model in which lentiviral vectors carrying the transgenes for human TNF and interferon (IFN)-γ were injected into the subarachnoid space to induce persistent cytokine production by meningeal cells, we have previously shown that persistently increased TNF and IFNγ in the CSF is sufficient to induce neuronal loss along with upregulation of TNFR1 and pMLKL in the areas with extensive inflammation [46]. This further supports our hypothesis that TNF-mediated inflammation, be it in vitro, in vivo, or during pathological conditions within the human brain, is sufficient to induce cell death through necroptosis activation.

In addition to the TNFR1 signaling, TNF has been reported to mediate Aβ-induced activation of dsRNA-dependent protein kinase (PKR), which underlies the shared pathogenic mechanisms between AD and type-II diabetes [33]. In recent years, studies have connected phosphorylation of RIPK1 with upstream PKR-signaling [24], suggesting yet another pathway by which TNF could trigger RIPK1 phosphorylation and subsequent necroptosis in the context of AD. In addition to the direct stimulation of necroptosis by TNF, this pro-inflammatory cytokine may also play a role in neurodegeneration by stimulating ROS generation in neurons and glia [11, 44, 52] and promoting glutamate release by microglia [14, 58]. In addition to the reduction in cell death and pMLKL expression, pre-treatment with RIPK1, RIPK3 and MLKL small molecule inhibitors also resulted in reduced beading of neurites, indicative of reduced neurodegeneration, and reduced intracellular ROS caused by TNF signaling. The latter is of significance as studies have shown that ROS functions in a positive feedback loop to ensure effective necroptosis activation, especially in the presence of TNF and a SMAC mimetic [53]. TNF can induce mitochondrial ROS, although ROS involvement in necroptosis is suggested to be cell context dependent [17, 20]. ROS can activate RIPK1 autophosphorylation and is essential for RIPK3 recruitment into the necrosome, thus enhancing necrosome formation. Conversely, ROS induction requires necrosomal RIPK3 [74]. Our data supports the role of ROS as a critical regulator of necroptosis in the context of TSZ treatment and the significant reduction of intracellular ROS by pre-treatment with necroptosis inhibitors correlates with increased cell survival.

ESCRT-III proteins, which are involved in endosomal trafficking, virus budding, and multivesicular body formation [47], have been shown to act downstream of MLKL, antagonizing necroptosis stimulated by TNF in vitro by extending the duration of plasma membrane integrity and sustaining cell survival [16]. Among the ESCRT-III components, mutations in the Chmp2B gene are reported to be associated with frontotemporal dementia (FTD) and amyotrophic lateral sclerosis (ALS) [64], which provides a clear link between dysfunction in the ESCRT-III machinery and neurodegeneration. Moreover, Chmp2B has been considered to be a GVD marker, as it shows strong immunoreactivity in the GVD bodies in AD brain [69]. However, to date there have been no reported associations between AD and known mutations of any ESCRT-III pathway components. Our data showed an increase in Chmp2B expression in the AD brain, specifically in the GVD bodies, and also in human iPSC-derived glutamatergic neurons treated with TSZ. Chmp2B expression also colocalized with pMLKL expression in the AD brain, thus confirming the presence of pMLKL in the GVD bodies. Although most studies have focused on the effects of overexpression of mutated Chmp2B (truncated) relevant to FTS and ALS, one study showed that overexpression of full-length Chmp2B in HeLa cells caused deformation of the plasma membrane [3]. Among other components, there was an increase in the expression of Vps24/Chmp3 in the AD brain, but not in cultured neurons. Chmp3 is required for the protein sorting and formation of multivesicular bodies (MVB) [2]. A constant recycling of Chmp3 by Vps4 is required to promote the net growth of ESCRT-III assemblies [37], and an overall increase in the levels of Chmp3 in the AD brain might indicate decreased turnover of Chmp3. A recent study showed that MAPT/Tau accumulation disrupts ESCRT-III complex formation in mice, thus impairing lysosomal degradation [10], which might explain the link between pTau expression and necroptosis in neurons, as shown in our study and others [30]. It has also been reported that MLKL and pMLKL levels can affect endosomal transport independently of RIPK3 and are required for the generation of intraluminal and extracellular vesicles [70]. Thus, the lack of effective extrusion of pMLKL from the plasma membrane into endosomes or exosomes may be responsible for vulnerability of certain cells to necroptosis. However, given the higher-than-expected number of neurons in the AD brain showing necroptosis markers along with ESCRT-III components, and the heterogeneity in the number of pMLKL positive puncta across these neurons, it could mean that they are trying to overcome necroptosis, and some will survive the insult. A subset of them may then fail to overcome the necroptotic signaling in the presence of additional factors such as pTau or proximity to higher levels of inflammatory mediators. Therefore, whether the increase in Chmp2B and Vps24 expression in the AD brain is the result of a compensatory mechanism that has been triggered in response to necroptosis to help neurons survive or is a dysregulation of their function that then results in failure to protect the neurons from necroptotic cell death is unclear. Detailed studies will be required to establish functional link between necroptosis and the ESCRT III pathway in neurons in vivo under pathological conditions.

In conclusion, we provide strong evidence for inflammation-driven activation of TNF-mediated necroptosis in CA1-2 hippocampal neurons in the post-mortem AD brain and propose that targeting the necroptosis pathway could hold great promise for inhibiting neurodegeneration. Our data suggests that certain neurons are more susceptible to necroptotic cell death, and that this selective neuronal vulnerability may be due to an inflammatory microenvironment contributed by activated microglia or CD8 + T cells that secrete proinflammatory cytokines, or due to dysregulation/imbalance in the ESCRT-III pathway or increased co-expression of pTau within those neurons. In addition, our data suggests gender-based differences that could also contribute to increased neuronal vulnerability to necroptosis in female brains in AD. Whether these gender-based differences are directly linked to differences in TNF signaling or may also involve other pathways remains to be determined. We hypothesize that the cascade of molecular pathways leading to the death of neurons by necroptosis could be an early event in the disease progression. While our results indicate that TNF-TNFR1 interaction may be a key mechanism that drives neurodegeneration in AD, it is important to note that there are other triggers and pathways leading to necroptotic cell death such as FasL, TRAIL, dsRNA/PKR, and viral Z-RNA [28, 41, 73], although their role in neuronal loss in AD is yet to be investigated. Emerging evidence of necroptosis in several neurodegenerative diseases and our own studies in MS and AD suggests convergence of molecular mechanisms underlying neuronal loss in different pathologies. Identifying the precise mechanisms underlying the loss of neurons in AD is important for developing effective strategies for the treatment of AD. Activation of RIPK1 has been demonstrated to mediate the majority of the pro-inflammatory and cell death inducing signaling stimulated by TNF/TNFR1 interaction, including those in the CNS [71]. RIPK1 is a key mediator of cell death and small molecule inhibitors of RIPK1 have advanced into clinical trials for several CNS and non-CNS disorders [18, 38]. Using small molecule inhibitors against RIPK1 and or other necroptosis pathway components in a suitable chronic animal model for AD showing neuronal loss would, therefore, provide definitive proof of a role for TNF/TNFR1 stimulated necroptosis in AD.

## Supplementary Information

**Additional file 1: Fig 1**. Cellular localization and gender difference in pMLKL and pRIPK3 expression.

**Additional file 2: Fig 2**. Association between pMLKL/pRIPK3 cell densities with AD pathology.

**Additional file 3: Fig 3**. mRNA analysis of ESCRT III pathway genes.

**Additional file 4: Fig 4**. Characterisation of human iPSC-derived glutamatergic neurons under different treatment conditions.

**Additional file 5: Fig 5**. Expression of ESCRT III components in response to TSZ treatment.

**Additional file 6: Fig 6**. Full blots of key proteins from TNF, necroptosis, apoptosis and ESCRT III pathways.

**Additional file 7: Table 1**. Clinical characteristics of the study cohort.

**Additional file 8: Table 2**. Antibody list.

## References

[1] Ashkenazi A, Salvesen G (2014). Regulated cell death: signaling and mechanisms. Annu Rev Cell Dev Biol.

[2] Babst M, Katzmann DJ, Estepa-Sabal EJ, Meerloo T, Emr SD (2002). Escrt-III: an endosome-associated heterooligomeric protein complex required for mvb sorting. Dev Cell.

[3] Bodon G, Chassefeyre R, Pernet-Gallay K, Martinelli N, Effantin G, Hulsik DL (2011). Charged multivesicular body protein 2B (CHMP2B) of the endosomal sorting complex required for transport-III (ESCRT-III) polymerizes into helical structures deforming the plasma membrane. J Biol Chem.

[4] Braak H, Braak E (1991). Neuropathological stageing of Alzheimer-related changes. Acta Neuropathol.

[5] Caccamo A, Branca C, Piras IS, Ferreira E, Huentelman MJ, Liang WS (2017). Necroptosis activation in Alzheimer's disease. Nat Neurosci.

[6] Calabrese M, Magliozzi R, Ciccarelli O, Geurts JJ, Reynolds R (2015). Exploring the origins of grey matter damage in multiple sclerosis. Nat Rev Neurosci.

[7] Declercq W, Vanden Berghe T, Vandenabeele P (2009). RIP kinases at the crossroads of cell death and survival. Cell.

[8] Dhawan G, Floden AM, Combs CK (2012). Amyloid-β oligomers stimulate microglia through a tyrosine kinase dependent mechanism. Neurobiol Aging.

[9] Durrenberger PF, Fernando FS, Magliozzi R, Kashefi SN, Bonnert TP, Ferrer I (2012). Selection of novel reference genes for use in the human central nervous system: a BrainNet Europe Study. Acta Neuropathol.

[10] Feng Q, Luo Y, Zhang XN, Yang XF, Hong XY, Sun DS (2020). MAPT/Tau accumulation represses autophagy flux by disrupting IST1-regulated ESCRT-III complex formation: a vicious cycle in Alzheimer neurodegeneration. Autophagy.

[11] Fischer R, Maier O (2015). Interrelation of oxidative stress and inflammation in neurodegenerative disease: role of TNF. Oxid Med Cell Longev.

[12] Fischer R, Kontermann RE, Pfizenmaier K (2020). Selective targeting of TNF receptors as a novel therapeutic approach. Front Cell Dev Biol.

[13] Fritsch M, Günther SD, Schwarzer R, Albert MC, Schorn F, Werthenbach JP (2019). Caspase-8 is the molecular switch for apoptosis, necroptosis and pyroptosis. Nature.

[14] Gallego-Delgado P, James R, Browne E (2020). Neuroinflammation in the normal-appearing white matter (NAWM) of the multiple sclerosis brain causes abnormalities at the nodes of Ranvier. PLoS Biol.

[15] Gate D, Saligrama N, Leventhal O, Yang AC, Unger MS, Middeldorp J (2020). Clonally expanded CD8 T cells patrol the cerebrospinal fluid in Alzheimer's disease. Nature.

[16] Gong YN, Guy C, Olauson H, Becker JU, Yang M, Fitzgerald P (2017). ESCRT-III acts downstream of MLKL to regulate necroptotic cell death and its consequences. Cell.

[17] Goossens V, De Vos K, Vercammen D, Steemans M, Vancompernolle K, Fiers W (1999). Redox regulation of TNF signaling. BioFactors.

[18] Grievink HW, Heuberger JAAC, Huang F, Chaudhary R, Birkhoff WAJ, Tonn GR (2020). DNL104, a centrally penetrant RIPK1 inhibitor, inhibits RIP1 kinase phosphorylation in a randomized phase i ascending dose study in healthy volunteers. Clin Pharmacol Ther.

[19] Han J, Zhong CQ, Zhang DW (2011). Programmed necrosis: backup to and competitor with apoptosis in the immune system. Nat Immunol.

[20] He S, Wang L, Miao L, Wang T, Du F, Zhao L, Wang X (2009). Receptor interacting protein kinase-3 determines cellular necrotic response to TNF-alpha. Cell.

[21] Heneka MT, Carson MJ, El Khoury J, Landreth GE, Brosseron F, Feinstein DL (2015). Neuroinflammation in Alzheimer's disease. Lancet Neurol.

[22] Hoffmann JC, Pappa A, Krammer PH, Lavrik IN (2009). A new C-terminal cleavage product of procaspase-8, p30, defines an alternative pathway of procaspase-8 activation. Mol Cell Biol.

[23] Hu WT, Howell JC, Ozturk T, Gangishetti U, Kollhoff AL, Hatcher-Martin JM (2019). CSF cytokines in aging, multiple sclerosis, and dementia. Front Immunol.

[24] Hugon J, Paquet C (2021). The PKR/P38/RIPK1 signaling pathway as a therapeutic target in Alzheimer's disease. Int J Mol Sci.

[25] Janelsins MC, Mastrangelo MA, Park KM, Sudol KL, Narrow WC, Oddo S (2008). Chronic neuron-specific tumor necrosis factor-alpha expression enhances the local inflammatory environment ultimately leading to neuronal death in 3xTg-AD mice. Am J Pathol.

[26] Jellinger KA, Stadelmann C (2001). Problems of cell death in neurodegeneration and Alzheimer's Disease. J Alzheimers Dis.

[27] Kaiser WJ, Upton JW, Long AB, Livingston-Rosanoff D, Daley-Bauer LP, Hakem R (2011). RIP3 mediates the embryonic lethality of caspase-8-deficient mice. Nature.

[28] Kearney CJ, Martin SJ (2017). An inflammatory perspective on necroptosis. Mol Cell.

[29] Kodama L, Guzman E, Etchegaray JI, Li Y, Sayed FA, Zhou L (2020). Microglial microRNAs mediate sex-specific responses to tau pathology. Nat Neurosci.

[30] Koper MJ, Van Schoor E, Ospitalieri S, Vandenberghe R, Vandenbulcke M, von Arnim CAF (2020). Necrosome complex detected in granulovacuolar degeneration is associated with neuronal loss in Alzheimer's disease. Acta Neuropathol.

[31] Lalaoui N, Boyden SE, Oda H, Wood GM, Stone DL, Chau D (2020). Mutations that prevent caspase cleavage of RIPK1 cause autoinflammatory disease. Nature.

[32] Leng F, Edison P (2021). Neuroinflammation and microglial activation in Alzheimer disease: Where do we go from here?. Nat Rev Neurol.

[33] Lourenco MV, Clarke JR, Frozza RL, Bomfim TR, Forny-Germano L, Batista AF (2013). TNF-α mediates PKR-dependent memory impairment and brain IRS-1 inhibition induced by Alzheimer's β-amyloid oligomers in mice and monkeys. Cell Metab.

[34] Magliozzi R, Howell OW, Durrenberger P, Aricò E, James R, Cruciani C (2019). Meningeal inflammation changes the balance of TNF signalling in cortical grey matter in multiple sclerosis. J Neuroinflammation.

[35] Martínez-Pinilla E, Ordóñez C, Del Valle E, Navarro A, Tolivia J (2016). Regional and gender study of neuronal density in brain during aging and in alzheimer's disease. Front Aging Neurosci.

[36] Meraz-Ríos MA, Toral-Rios D, Franco-Bocanegra D, Villeda-Hernández J, Campos-Peña V (2013). Inflammatory process in Alzheimer's disease. Front Integr Neurosci.

[37] Mierzwa BE, Chiaruttini N, Redondo-Morata L, von Filseck JM, König J, Larios J (2017). Dynamic subunit turnover in ESCRT-III assemblies is regulated by Vps4 to mediate membrane remodelling during cytokinesis. Nat Cell Biol.

[38] Mifflin L, Ofengeim D, Yuan J (2020). Receptor-interacting protein kinase 1 (RIPK1) as a therapeutic target. Nat Rev Drug Discov.

[39] Mirra SS, Heyman A, McKeel D, Sumi SM, Crain BJ, Brownlee LM (1991). The consortium to establish a registry for Alzheimer’s disease (CERAD): part II Standardization of the neuropathologic assessment of Alzheimer’s disease. Neurology.

[40] Moquin DM, McQuade T, Chan FK (2013). CYLD deubiquitinates RIP1 in the TNFα-induced necrosome to facilitate kinase activation and programmed necrosis. PLoS ONE.

[41] Murakami Y, Matsumoto H, Roh M, Giani A, Kataoka K, Morizane Y (2014). Programmed necrosis, not apoptosis, is a key mediator of cell loss and DAMP-mediated inflammation in dsRNA-induced retinal degeneration. Cell Death Differ.

[42] Ofengeim D, Yuan J (2013). Regulation of RIP1 kinase signalling at the crossroads of inflammation and cell death. Nat Rev Mol Cell Biol.

[43] Ofengeim D, Ito Y, Najafov A, Zhang Y, Shan B, DeWitt JP (2015). Activation of necroptosis in multiple sclerosis. Cell Rep.

[44] Olmos G, Lladó J (2014). Tumor necrosis factor alpha: a link between neuroinflammation and excitotoxicity. Mediators Inflamm.

[45] Park J, Ha HJ, Chung ES, Baek SH, Cho Y, Kim HK (2021). O-GlcNAcylation ameliorates the pathological manifestations of Alzheimer's disease by inhibiting necroptosis. Sci Adv.

[46] Picon C, Jayaraman A, James R, Beck C, Gallego P, Witte ME (2021). Neuron-specific activation of necroptosis signaling in multiple sclerosis cortical grey matter. Acta Neuropathol.

[47] Raiborg C, Stenmark H (2009). The ESCRT machinery in endosomal sorting of ubiquitylated membrane proteins. Nature.

[48] Re DB, Le Verche V, Yu C, Amoroso MW, Politi KA, Phani S (2014). Necroptosis drives motor neuron death in models of both sporadic and familial ALS. Neuron.

[49] Rehker J, Rodhe J, Nesbitt RR, Boyle EA, Martin BK, Lord J (2017). Caspase-8, association with Alzheimer's Disease and functional analysis of rare variants. PLoS ONE.

[50] Rosario ER, Chang L, Head EH, Stanczyk FZ, Pike CJ (2009). Brain levels of sex steroid hormones in men and women during normal aging and in Alzheimer's disease. Neurobiol Aging.

[51] Roth KA (2001). Caspases, apoptosis, and alzheimer disease: causation, correlation, and confusion. J Neuropathol Exp Neurol.

[52] Sandoval R, Lazcano P, Ferrari F, Pinto-Pardo N, González-Billault C, Utreras E (2018). TNF-α increases production of reactive oxygen species through Cdk5 activation in nociceptive neurons. Front Physiol.

[53] Schenk B, Fulda S (2015). Reactive oxygen species regulate Smac mimetic/TNFα-induced necroptotic signaling and cell death. Oncogene.

[54] Schmittgen TD, Livak KJ (2008). Analyzing real-time PCR data by the comparative C(T) method. Nat Protoc.

[55] Sharma VK, Singh TG, Singh S, Garg N, Dhiman S (2021). Apoptotic pathways and Alzheimer's disease: probing therapeutic potential. Neurochem Res.

[56] Silke J, Strasser A (2013). The FLIP side of life. Sci Signal.

[57] Skup M, Zhu H, Wang Y, Giovanello KS, Lin JA, Shen D (2011). Alzheimer's Disease Neuroimaging Initiative. Sex differences in grey matter atrophy patterns among AD and aMCI patients: results from ADNI. Neuroimage.

[58] Socodato R, Portugal CC, Canedo T, Rodrigues A, Almeida TO, Henriques JF (2020). Microglia dysfunction caused by the loss of rhoa disrupts neuronal physiology and leads to neurodegeneration. Cell Rep.

[59] Stojić-Vukanić Z, Hadžibegović S, Nicole O, Nacka-Aleksić M, Leštarević S, Leposavić G (2020). CD8+ T Cell-mediated mechanisms contribute to the progression of neurocognitive impairment in both multiple sclerosis and Alzheimer's disease?. Front Immunol.

[60] Sun L, Wang H, Wang Z, He S, Chen S, Liao D (2012). Mixed lineage kinase domain-like protein mediates necrosis signaling downstream of RIP3 kinase. Cell.

[61] Swardfager W, Lanctôt K, Rothenburg L, Wong A, Cappell J, Herrmann N (2010). A meta-analysis of cytokines in Alzheimer's disease. Biol Psychiatry.

[62] Tarkowski E, Andreasen N, Tarkowski A, Blennow K (2003). Intrathecal inflammation precedes development of Alzheimer's disease. J Neurol Neurosurg Psychiatry.

[63] Toro CA, Zhang L, Cao J, Cai D (2019). Sex differences in Alzheimer's disease: understanding the molecular impact. Brain Res.

[64] Ugbode C, West RJH (2021). Lessons learned from CHMP2B, implications for frontotemporal dementia and amyotrophic lateral sclerosis. Neurobiol Dis.

[65] Vandenabeele P, Galluzzi L, Vanden Berghe T, Kroemer G (2010). Molecular mechanisms of necroptosis: an ordered cellular explosion. Nat Rev Mol Cell Biol.

[66] Walker DG, Lue LF (2015). Immune phenotypes of microglia in human neurodegenerative disease: challenges to detecting microglial polarization in human brains. Alz Res Therapy.

[67] Wang T (2015). TNF-alpha G308A polymorphism and the susceptibility to Alzheimer's disease: an updated meta-analysis. Arch Med Res.

[68] Weber K, Roelandt R, Bruggeman I, Estornes Y, Vandenabeele P (2018). Nuclear RIPK3 and MLKL contribute to cytosolic necrosome formation and necroptosis. Commun Biol.

[69] Yamazaki Y, Takahashi T, Hiji M, Kurashige T, Izumi Y, Yamawaki T, Matsumoto M (2010). Immunopositivity for ESCRT-III subunit CHMP2B in granulovacuolar degeneration of neurons in the Alzheimer's disease hippocampus. Neurosci Lett.

[70] Yoon S, Kovalenko A, Bogdanov K, Wallach D (2017). MLKL, the Protein that mediates necroptosis, also regulates endosomal trafficking and extracellular vesicle generation. Immunity.

[71] Yuan J, Amin P, Ofengeim D (2019). Necroptosis and RIPK1-mediated neuroinflammation in CNS diseases. Nat Rev Neurosci.

[72] Zhang B, Gaiteri C, Bodea L-G, Wang Z (2013). Integrated systems approach identifies genetic nodes and networks in late-onset Alzheimer's disease. Cell.

[73] Zhang T, Yin C, Boyd DF, Quarato G, Ingram JP, Shubina M (2020). Influenza virus Z-RNAs induce ZBP1-mediated necroptosis. Cell.

[74] Zhang Y, Su SS, Zhao S, Yang Z, Zhong CQ, Chen X (2017). RIP1 autophosphorylation is promoted by mitochondrial ROS and is essential for RIP3 recruitment into necrosome. Nat Commun.

[75] Zhao M, Cribbs DH, Anderson AJ, Cummings BJ, Su JH, Wasserman AJ, Cotman CW (2003). The induction of the TNFalpha death domain signaling pathway in Alzheimer's disease brain. Neurochem Res.

[76] Zhou W, Yuan J (2014). Necroptosis in health and diseases. Semin Cell Dev Biol.

