# Supplementary figures and images for: TNF-mediated neuroinflammation is linked to neuronal necroptosis in Alzheimer's disease hippocampus
Source: Acta Neuropathol Commun. 2021 Sep 28;9:159. doi: 10.1186/s40478-021-01264-w (PMC8501605; doi:10.1186/s40478-021-01264-w)

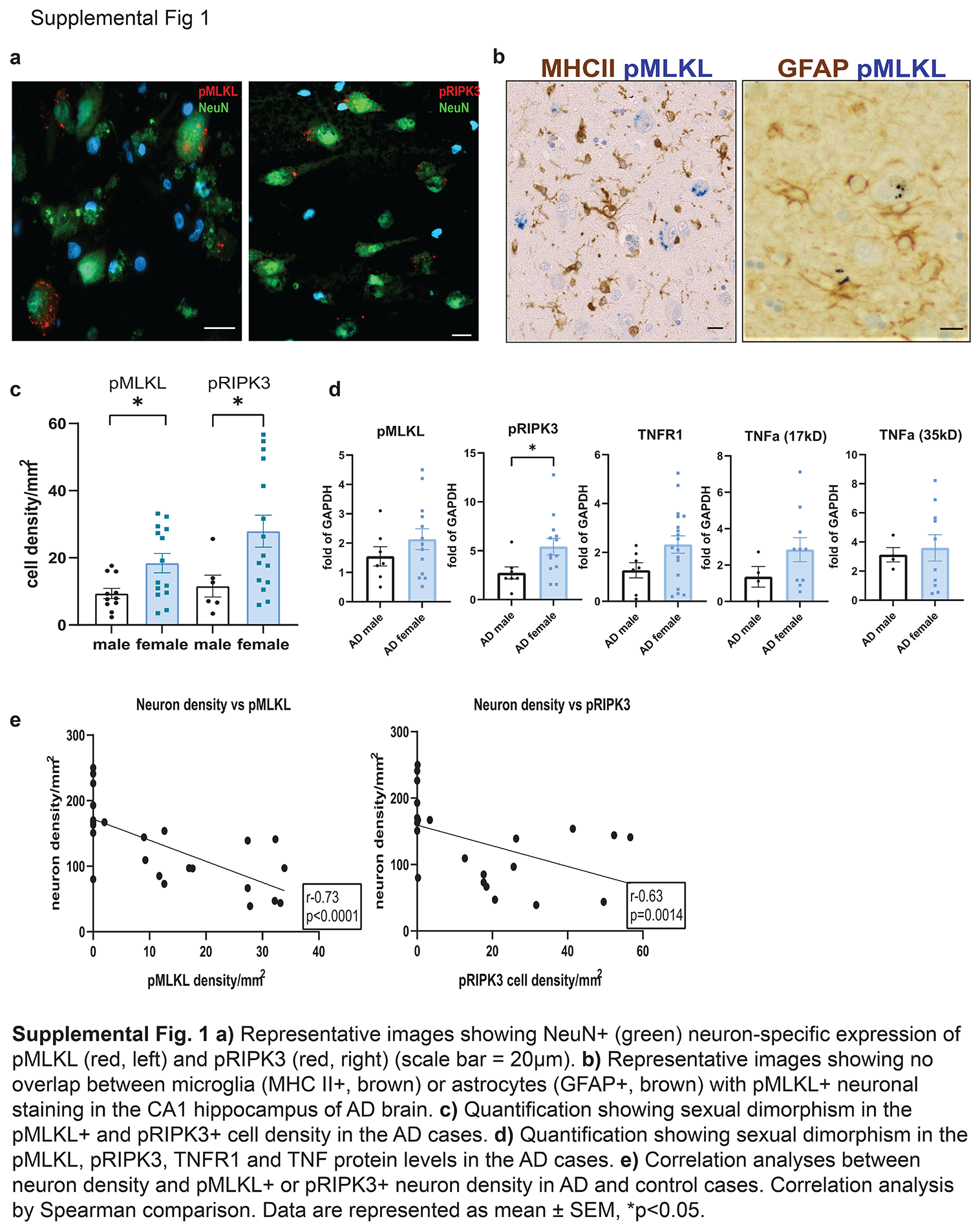

Supplement: Supplementary file 1 — Additional file 1: Fig 1. Cellular localization and gender difference in pMLKL and pRIPK3 expression. [file 40478_2021_1264_MOESM1_ESM.tif]

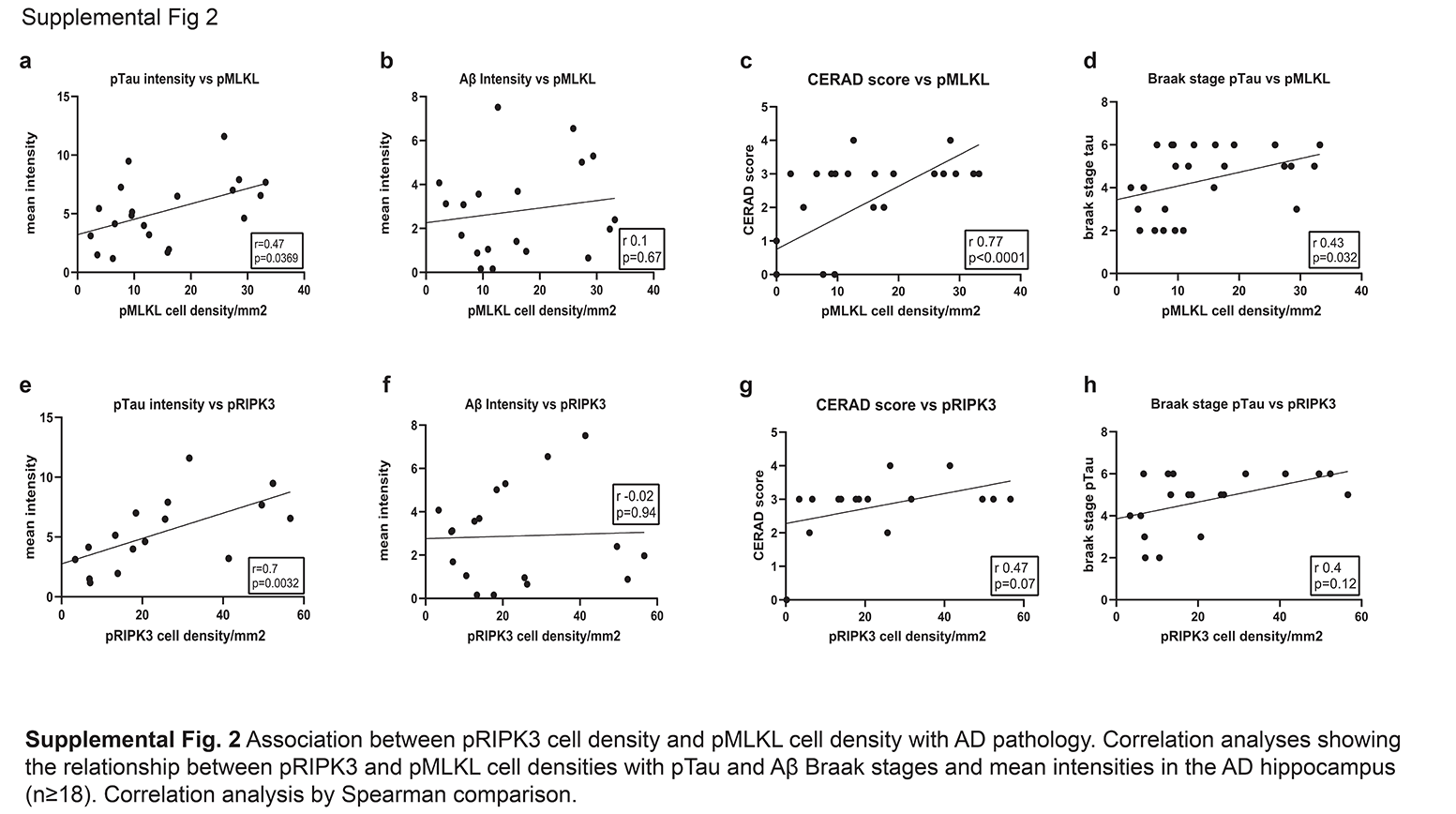

Supplement: Supplementary file 2 — Additional file 2: Fig 2. Association between pMLKL/pRIPK3 cell densities with AD pathology. [file 40478_2021_1264_MOESM2_ESM.tif]

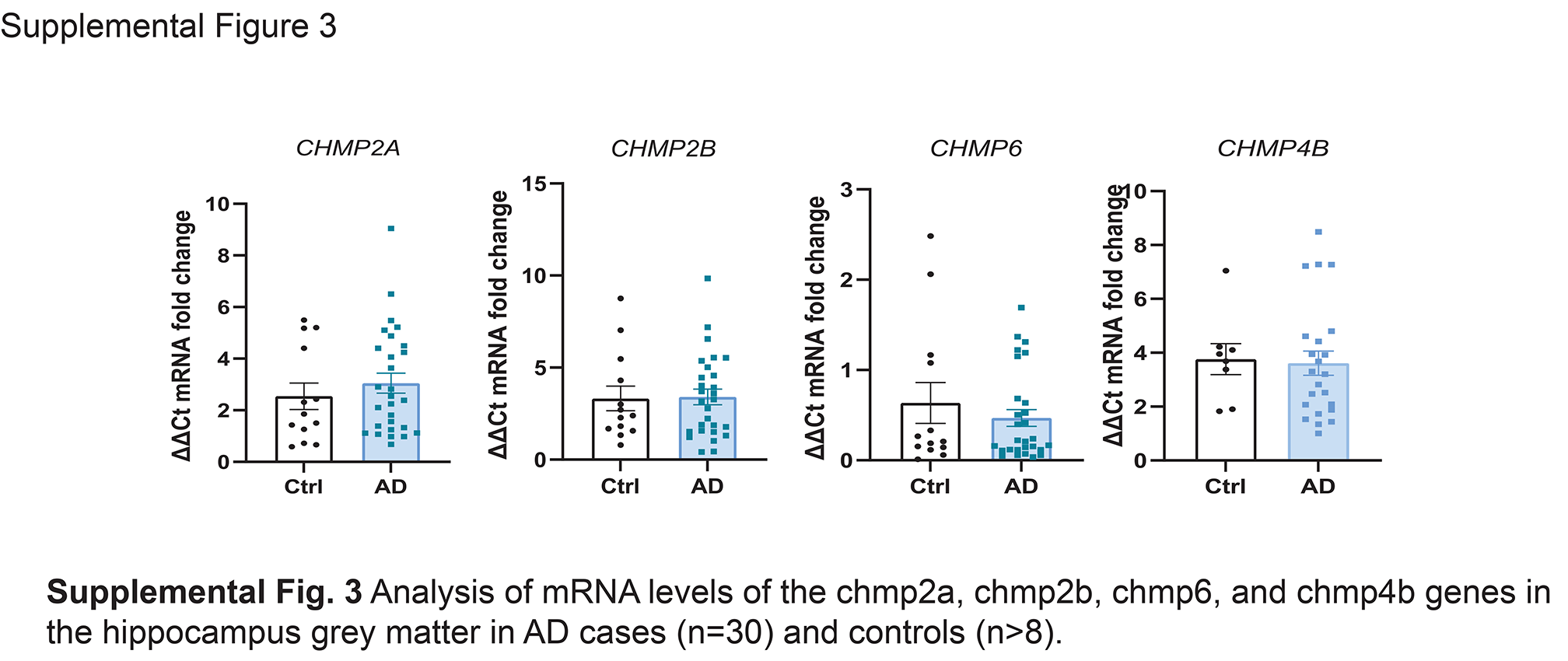

Supplement: Supplementary file 3 — Additional file 3: Fig 3. mRNA analysis of ESCRT III pathway genes. [file 40478_2021_1264_MOESM3_ESM.tif]

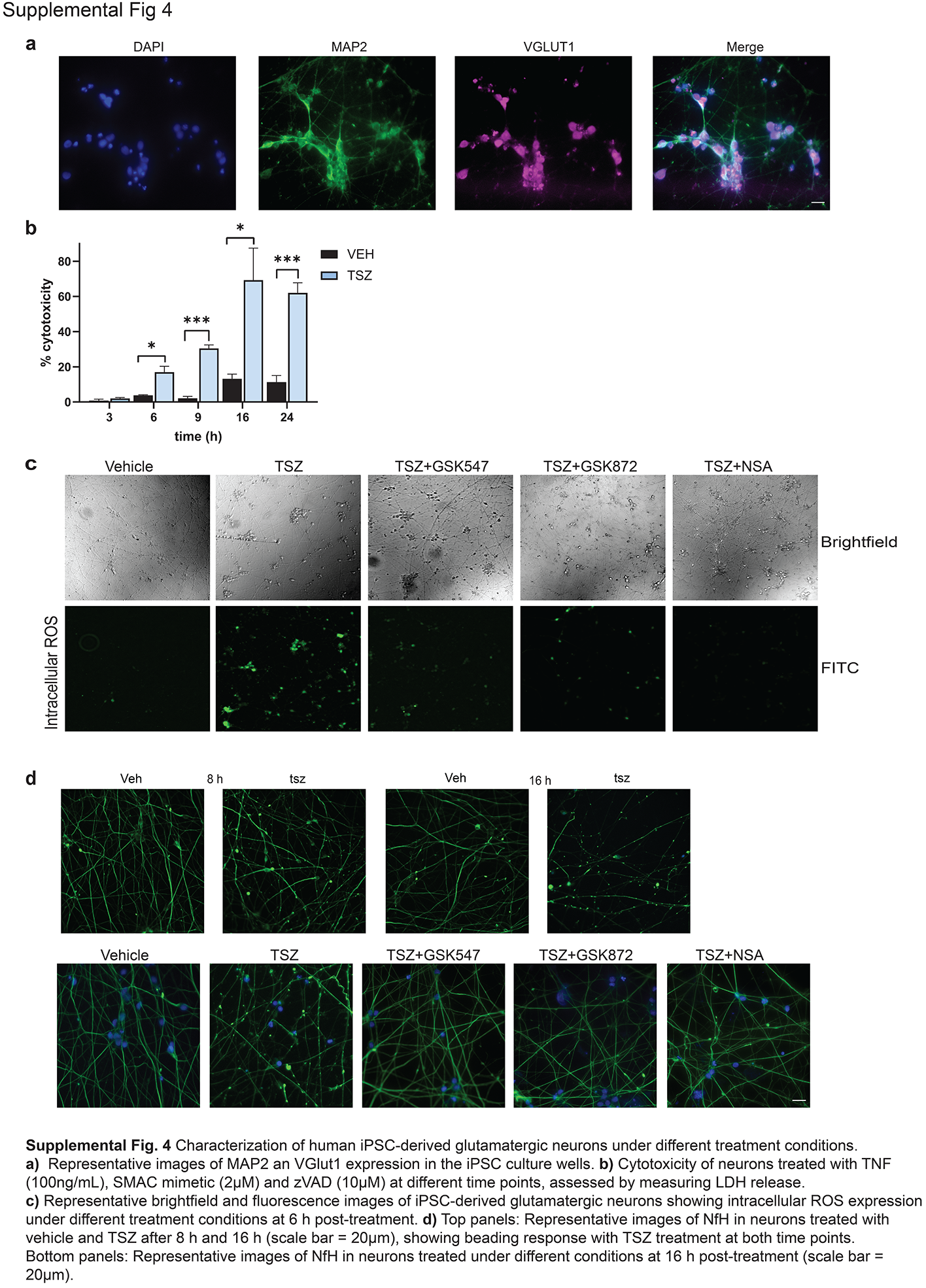

Supplement: Supplementary file 4 — Additional file 4: Fig 4. Characterisation of human iPSC-derived glutamatergic neurons under different treatment conditions. [file 40478_2021_1264_MOESM4_ESM.tif]

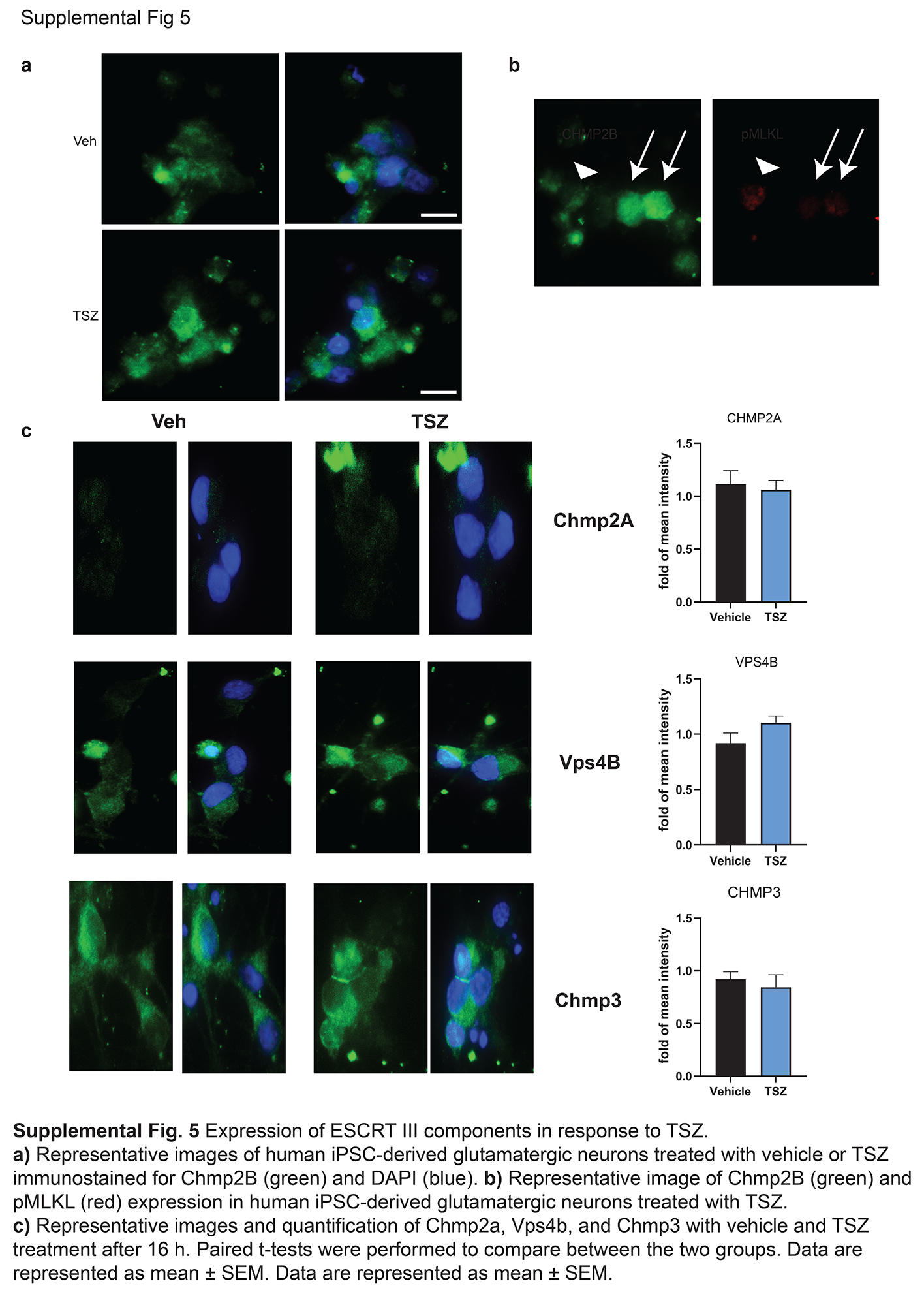

Supplement: Supplementary file 5 — Additional file 5: Fig 5. Expression of ESCRT III components in response to TSZ treatment. [file 40478_2021_1264_MOESM5_ESM.tif]

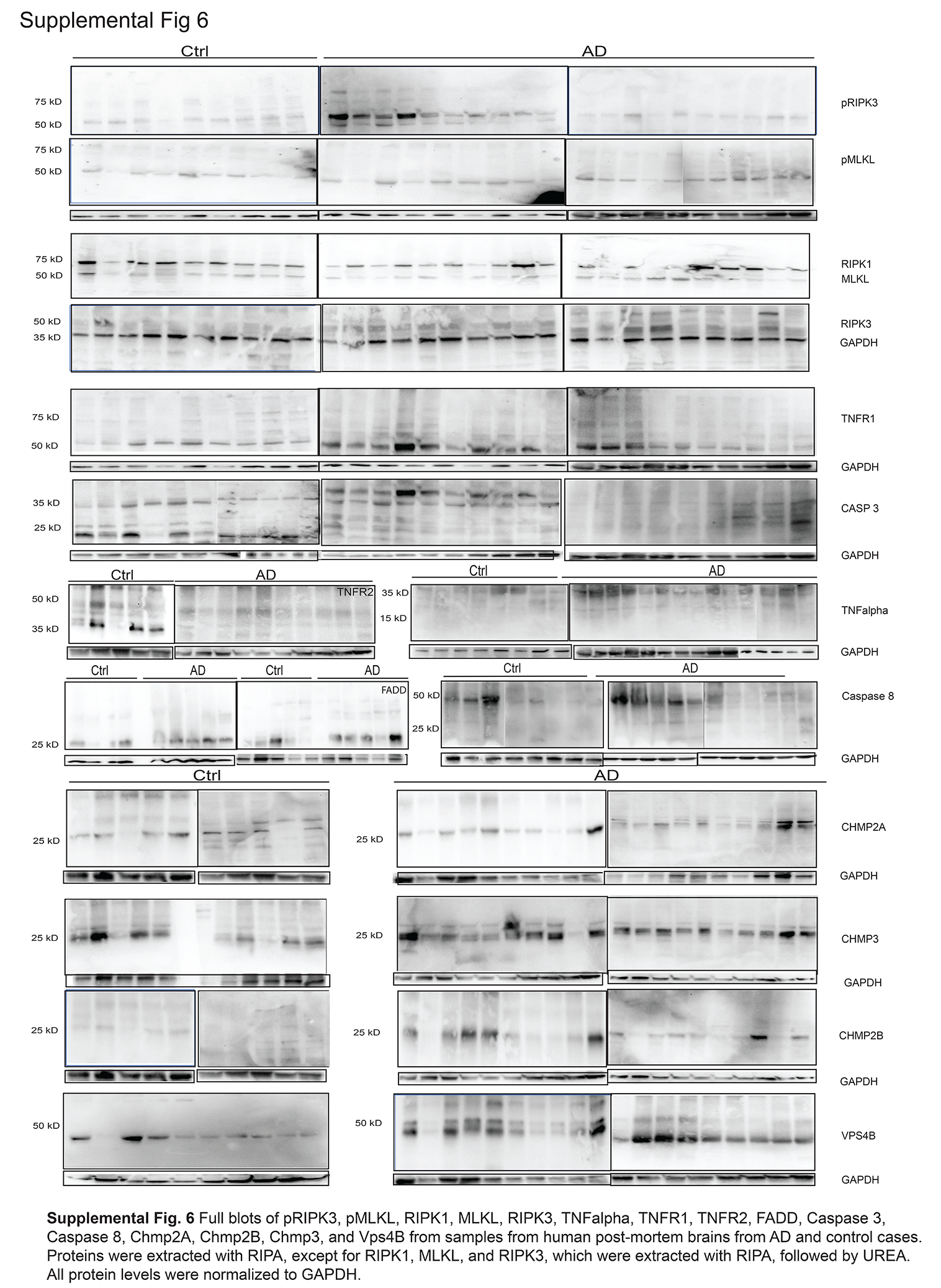

Supplement: Supplementary file 6 — Additional file 6: Fig 6. Full blots of key proteins from TNF, necroptosis, apoptosis and ESCRT III pathways. [file 40478_2021_1264_MOESM6_ESM.tif]
