# Supplementary material for: TNF-mediated neuroinflammation is linked to neuronal necroptosis in Alzheimer's disease hippocampus
Source: Acta Neuropathol Commun. 2021 Sep 28;9:159. doi: 10.1186/s40478-021-01264-w (PMC8501605; doi:10.1186/s40478-021-01264-w)
Supplement: Supplementary file 7 — Additional file 7: Table 1. Clinical characteristics of the study cohort. [file 40478_2021_1264_MOESM7_ESM.docx]

Supplementary Table 1: Cohort characteristics

| Case number | Age  (years) | Sex | Braak stage^[4]^ | CERAD score^[39]^ | Clinical diagnosis | Post-mortem delay (h) |
| --- | --- | --- | --- | --- | --- | --- |
| 1  2  3  4  5  6  7  8  9  10  11  12  13  14  15  16  17  18  19  20  21  22  23  24  25  26  27  28  29  30  31  32  33  34  35  36  37  38  39  40  41 | 85  74  83  78  95  83  89  93  78  92  63  66  93  91  87  73  86  88  75  80  57  89  80  92  89  86  83  85  86  83  99  90  78  87  70  84  85  68  84  80  87 | M  F  M  M  F  M  F  M  F  M  F  F  F  F  F  F  M  F  F  M  F  F  M  F  F  F  M  F  M  M  F  M  F  M  M  F  M  M  F  F  M | 2  1  3  2  2  2  2  2  2  2  1  6  5  5  5  6  4  5  5  6  6  6  5  6  5  5  3  6  5  3  5  5  6  6  6  4  4  4  4  4  5 | 1  0  0  0  0  0  0  0  0  0  0  4  4  3  3  3  3  3  3  3  3  3  2  3  3  3  1  3  3  3  3  3  3  3  3  1  2  2  3  2  3 | Non-AD control  Non-AD control  Non-AD control, mild CAA  Non-AD control  Non-AD control  Non-AD control  Non-AD control  Non-AD control  Non-AD control  Non-AD control, CAA  Non-AD control  AD, CAA, Dementia  AD  AD, Dementia  AD  AD  AD, HS  AD, SVD, Dementia  AD, BA, VaD  AD, Dementia  AD, CAA, Early-onset Dementia  AD  AD  AD  AD, SVD, Dementia  AD  AD, MVD  AD. CAA  AD, VaD  AD, Dementia  AD, SVD, Dementia  AD  AD, Dementia  AD, CAA, SVD, Dementia  AD, Dementia  AD  AD  AD  AD, Dementia  AD  AD | 30.5  39.5  37.5  56.25  59.25  47.75  26.5  34.5  66.25  47.25  21  37.25  31.75  28.25  40.5  25.75  34.5  80.5  37.5  21.25  30.5  31.25  43  39.5  35.75  37.5  45.5  17.75  47.5  41.75  43  61  27.75  44.25  57  22  23  24  13.5  28  41.75 |

*AD* Alzheimer’s disease, *F* female, *M* male, *CAA* cerebrovascular angiopathy, *HS* hippocampal sclerosis, *SVD* subcortical vascular dementia, *BA* basal atherosclerosis, *MVD* moderate vascular disease, *VaD* vascular dementia. CERAD scores for neuritic plaque densities: 0 = none (no plaques), 1 = sparse, 2 = moderate, and 3 = High density.
