# Supplementary material for: TNF-mediated neuroinflammation is linked to neuronal necroptosis in Alzheimer's disease hippocampus
Source: Acta Neuropathol Commun. 2021 Sep 28;9:159. doi: 10.1186/s40478-021-01264-w (PMC8501605; doi:10.1186/s40478-021-01264-w)
Supplement: Supplementary file 8 — Additional file 8: Table 2. Antibody list. [file 40478_2021_1264_MOESM8_ESM.docx]

Supplementary Table 2: Antibody list

| **REAGENTS** | **SOURCE** | **IDENTIFIER** | **APPLICATION** |
| --- | --- | --- | --- |
| Mouse monoclonal anti-human PHF-Tau antibody | Thermo Scientific | Cat# MN1020  **RRID:AB_223647** | IF and IHC (1:500) |
| Mouse monoclonal anti-amyloid βA4 (1E8) | Millipore | Cat# MABN639 | IF and IHC (1:500) |
| Mouse monoclonal anti-GAPDH antibody | Bio-Rad | Cat#VMA00046 | WB (1:1000) |
| Mouse monoclonal anti-MLKL (32B) antibody | Santa Cruz Biotechnology | Cat#sc-293201 | WB (1:1000) |
| Rabbit polyclonal anti-phospho-human MLKL (Ser 358) antibody | Abcam | Cat# ab187091  **RRID:AB_2619685** | WB (1:1000); IHC and IF (1:200) |
| Mouse monoclonal anti- human RIPK3 antibody | R&D Systems | Cat# MAB7604  **RRID:AB_2619684** | WB (1:1000) |
| Rabbit monoclonal anti-phospho-human RIPK3 (Ser 227) antibody | Cell Signaling Technology | Cat# 93654  **RRID:AB_847259** | IHC and IF (1:250)  WB (1:1000) |
| Mouse monoclonal anti-RIPK1 (7H10) antibody | Abcam | Cat# ab72139  **RRID:AB_2178115** | WB (1:1000); IF (1:200) |
| Rabbit monoclonal anti-TNF receptor II antibody | Abcam | Cat#ab109322 | WB (1:1000) |
| Mouse monoclonal anti-NeuN antibody | Millipore | Cat# MAB377 | IF (1:1000) |
| Rabbit monoclonal anti-FADD antibody | Abcam | Cat# ab108601  **RRID:AB_10864812** | WB (1:1000) |
| Rabbit polyclonal anti-active caspase 3 antibody | R&D systems | Cat# AF835  **RRID:AB_2243952** | WB (1:1000)  IF (1:200) |
| Rabbit monoclonal anti-CD8 (SP16) antibody | Invitrogen | Cat# MA5-14548  **RRID:AB_10984334** | IHC (1:250) |
| Mouse monoclonal anti-caspase-8 (IC12) antibody | Cell Signaling Technology | Cat# 9746 | WB (1:1000) |
| Mouse monoclonal anti-HuC/D antibody (16A11) | Invitrogen | Cat# A-21271  **RRID:AB_221448** | IHC (1:500) |
| Mouse monoclonal anti-human TNFRI antibody | R and D Systems | Cat# MAB225  **RRID:AB_2204150** | WB (1:1000) |
| Mouse monoclonal anti-Neurofilament 200 kDa (RT97) | Millipore | Cat# MAB5262 | IF (1:1000) |
| Rabbit monoclonal anti-TNF alpha antibody | Abcam | Cat# ab215188 | WB (1:1000) |
| Rabbit polyclonal anti-TNFR1 antibody | Abcam | Cat# ab223352 | IF (1:100) |
| Rabbit polyclonal anti-VPS4B/MIG1 antibody | Abcam | Cat# ab224736 | WB (1:1000)  IF (1:200) |
| Mouse monoclonal anti-MAP2 antibody | Abcam | Cat# ab254143 | IF (1:50) |
| Rabbit monoclonal anti-VGlut1 antibody | Abcam | Cat# ab227805  **RRID:AB_2868428** | IF (1:500) |
| Rabbit monoclonal anti-VPS24 antibody | Abcam | Cat# ab175930 | WB (1:1000) |
| Rabbit monoclonal anti-CHMP2B antibody | Abcam | Cat# ab157208  **RRID:AB_2885096** | WB (1:1000)  IF (1:250) |
| Mouse monoclonal anti-human HLA-DR/DP/DQ (CR3/43) antibody | Thermo Scientific | Cat# MA1-25914  **RRID:AB_794857** | IHC (1:500) |
| Rabbit polyclonal anti-GFAP antibody | Abcam | Cat# ab7260  **RRID:AB_305808** | IHC (1:500) |
| ImmPACT® DAB Substrate, Peroxidase (HRP) | Vector Laboratories | Cat# SK-4105 |  |
| Vector® Blue Substrate Kit, Alkaline Phosphatase (AP) | Vector Laboratories | Cat# SK-5300 |  |
| VECTASTAIN® ABC-AP Kit, Alkaline Phosphatase | Vector Laboratories | AK-500 |  |
| ImmPRESS® HRP Horse Anti-Rabbit IgG Polymer Detection Kit, Peroxidase | Vector Laboratories | Cat# MP-7401, |  |
| ImmPRESS® HRP Horse Anti-Mouse IgG Polymer Detection Kit, Peroxidase | Vector Laboratories | Cat# MP-7422, |  |
| Goat anti-Rabbit IgG (H+L) Secondary Antibody, Alexa Fluor 488, preabsorbed | Abcam | Cat# ab150081  **RRID:AB_2734747** | IHC (1:5000) |
| Goat Anti-Mouse IgG (H+L) Antibody, Alexa Fluor 488, preabsorbed | Abcam | Cat# ab150117  **RRID:AB_2688012** | IHC (1:5000) |
| Goat anti-Mouse IgG (H+L) Secondary Antibody, Alexa Fluor 647, preabsorbed | Abcam | Cat# ab150119  **RRID:AB_2811129** | IF (1:2000) |
| Goat anti-Rabbit IgG (H+L) Secondary Antibody, Alexa Fluor 594, preabsorbed | Abcam | Cat# ab150084  **RRID:AB_2734147** | IF (1:2000) |
| Goat anti-Rabbit IgG (H+L) Secondary Antibody, Alexa Fluor 555, preabsorbed | Abcam | Cat# ab150086  **RRID:AB_2890032** | IHC (1:5000) |
| Goat anti-Mouse IgG (H+L) Secondary Antibody, Alexa Fluor 555, preabsorbed | Abcam | Cat# ab150118  **RRID:AB_2714033** | IHC (1:5000) |
| Goat anti-Mouse IgG (H/L) HRP | Bio-Rad | Cat# 0300-0108P | WB (1:20000) |
| Goat anti-Rabbit IgG HRP | Bio-Rad | Cat# 403005 | WB (1:20000) |
